# Apps for IMproving FITness and Increasing Physical Activity Among Young People: The AIMFIT Pragmatic Randomized Controlled Trial

**DOI:** 10.2196/jmir.4568

**Published:** 2015-08-27

**Authors:** Artur Direito, Yannan Jiang, Robyn Whittaker, Ralph Maddison

**Affiliations:** ^1^ Faculty of Medical and Health Sciences National Institute for Health Innovation University of Auckland Auckland New Zealand

**Keywords:** physical fitness, motor activity, exercise, physical activity, adolescent, health promotion, telemedicine, mHealth, mobile applications, smartphone

## Abstract

**Background:**

Given the global prevalence of insufficient physical activity (PA), effective interventions that attenuate age-related decline in PA levels are needed. Mobile phone interventions that positively affect health (mHealth) show promise; however, their impact on PA levels and fitness in young people is unclear and little is known about what makes a good mHealth app.

**Objective:**

The aim was to determine the effects of two commercially available smartphone apps (Zombies, Run and Get Running) on cardiorespiratory fitness and PA levels in insufficiently active healthy young people. A second aim was to identify the features of the app design that may contribute to improved fitness and PA levels.

**Methods:**

Apps for IMproving FITness (AIMFIT) was a 3-arm, parallel, randomized controlled trial conducted in Auckland, New Zealand. Participants were recruited through advertisements in electronic mailing lists, local newspapers, flyers posted in community locations, and presentations at schools. Eligible young people aged 14-17 years were allocated at random to 1 of 3 conditions: (1) use of an immersive app (Zombies, Run), (2) use of a nonimmersive app (Get Running), or (3) usual behavior (control). Both smartphone apps consisted of a fully automated 8-week training program designed to improve fitness and ability to run 5 km; however, the immersive app featured a game-themed design and narrative. Intention-to-treat analysis was performed using data collected face-to-face at baseline and 8 weeks, and all regression models were adjusted for baseline outcome value and gender. The primary outcome was cardiorespiratory fitness, objectively assessed as time to complete the 1-mile run/walk test at 8 weeks. Secondary outcomes were PA levels (accelerometry and self-reported), enjoyment, psychological need satisfaction, self-efficacy, and acceptability and usability of the apps.

**Results:**

A total of 51 participants were randomized to the immersive app intervention (n=17), nonimmersive app intervention (n=16), or the control group (n=18). The mean age of participants was 15.7 (SD 1.2) years; participants were mostly NZ Europeans (61%, 31/51) and 57% (29/51) were female. Overall retention rate was 96% (49/51). There was no significant intervention effect on the primary outcome using either of the apps. Compared to the control, time to complete the fitness test was –28.4 seconds shorter (95% CI –66.5 to 9.82, P=.20) for the immersive app group and –24.7 seconds (95% CI –63.5 to 14.2, P=.32) for the nonimmersive app group. No significant intervention effects were found for secondary outcomes.

**Conclusions:**

Although apps have the ability to increase reach at a low cost, our pragmatic approach using readily available commercial apps as a stand-alone instrument did not have a significant effect on fitness. However, interest in future use of PA apps is promising and highlights a potentially important role of these tools in a multifaceted approach to increase fitness, promote PA, and consequently reduce the adverse health outcomes associated with insufficient activity.

**Trial Registration:**

Australian New Zealand Clinical Trials Registry: ACTRN12613001030763; https://www.anzctr.org.au/Trial/Registration/TrialReview.aspx?ACTRN=12613001030763 (Archived by WebCite at http://www.webcitation.org/6aasfJVTJ).

## Introduction

Worldwide, 80.3% (95% CI 80.1-80.5) of adolescents aged 13 to 15 years do not achieve current physical activity (PA) recommendations [1]. New Zealand mirrors these data with 67.7% in the age group of 15 to 19 years not complying with these guidelines [2]. A recent analysis of 50 studies including more than 25 million fitness test results of young people aged 9 to 17 years from 28 countries reported global declines of cardiorespiratory fitness (CRF) of approximately 5% every decade from 1970 to 2000 [3]. Insufficient activity is associated with increased risk of chronic diseases, such as cardiovascular diseases, type 2 diabetes mellitus, some types of cancer, and premature death [4,5]. Likewise, lower levels of CRF, an important marker of overall physiologic health [6,7] sensitive to patterns of PA, also predict future morbidity and mortality [8,9]. Increasing levels of PA and CRF in young people are important public health priorities for enhancing health, well-being, and preventing disease [10].

Physical activity declines with age throughout adolescence [2,11,12] and tracks into adulthood [13], making this an important time to intervene. Common approaches for intervening in this population have included school-based programs because schools integrate almost all young people. A Cochrane systematic review of school-based PA interventions targeting areas such as curriculum, teacher training, educational materials, and accessibility to exercise equipment (44 randomized controlled trials [RCTs], N=36,593, age 6-18 years) provided some evidence of effectiveness for increasing time spent in moderate-to-vigorous intensity PA (MVPA) from 5 to 45 minutes more per day and CRF (VO_2peak_) from 1.6 to 3.7 mL/kg/min [14]. However, caution interpreting these findings is warranted because the included studies were at moderate risk of bias, with the majority having relied on self-reported activity [15]. Notably, a 2012 meta-analysis (30 RCTs, N=14,326, age ≤16 years), which differs from previous systematic reviews [16] in that it only included studies with accelerometry-measured outcomes (ie, objectively measured), showed small treatment effects for both total PA (standardized mean difference 0.12, 95% CI 0.04-0.20, *P*<.01) and MVPA (standardized mean difference 0.16, 95% CI 0.08-0.24, *P*<.001) [17]. These results translate into approximately 4 minutes more walking or running per day, a modest increase with limited clinical significance.

Many existing interventions, including school-based interventions, are limited because they are resource intensive. Young people are considered a hard-to-reach group and typically have limited adherence or exposure to PA interventions [16]. Therefore, novel approaches are crucial to engage those who are insufficiently active to encourage good health [18]. Given young peoples’ ubiquitous use of mobile phones and increased digital literacy [19,20], interventions leveraging this technology may provide a promising intervention for this population.

Young people are early adopters of new consumer technology, such as smartphones and apps. Smartphone ownership among young people is on the rise, having increased from 23% in 2011 to 37% in 2012 in the United States, with similar distribution across ethnicity and family income [21]. Moreover, 58% of all US young people aged 12 to 17 years reported having downloaded apps compared to 34% of adults [22]. Likewise, digital divides have decreased between 2007 and 2013 in New Zealand [23], with 75% of high-income families reporting household access to a smartphone compared with 52% for those with lower income [24]. Alongside the increased ownership of smartphones, there has been a proliferation of apps in the major platform operators’ app stores, which currently offer more than 100,000 mHealth apps [25]. Consumers looking for help via their smartphone face an immense number of mHealth apps [26] and the majority of these (31%) are fitness apps [25]. This high engagement with mobile technology offers an ideal opportunity to leverage the benefits of mobile interventions for health (mHealth), including lower participant burden and flexibility [27].

The effectiveness of mHealth-delivered interventions to promote PA that use texting or short message service (SMS) [28,29], personal digital assistants (PDAs) [30], and apps in addition to other components [31] has been examined. Unlike SMS text messaging, which has a substantial body of literature supporting its use, more complex interventions that capitalize on the computational power of smartphones have only recently been developed. Particularly, few studies have used an entirely mHealth device-based approach to deliver health behavior change interventions [32]. Most [33,34] include other components, such as a website, individual face-to-face or group sessions, phone calls, print materials, or pedometers, making it impossible to tease out the specific effects of each component. Thus, at present, there is a lack of scientific evidence assessing publicly available apps to promote PA. Because these apps are commercially available to the public on app stores and because fitness apps are currently the most popular (78% of users in 2014 compared to 39% in 2013 [35]), it is important to determine whether popular/commonly downloaded apps are effective [36].

Therefore, the primary aim of the Apps for IMproving FITness (AIMFIT) trial was to evaluate the effectiveness of two popular commercially available smartphone apps [37] for improving CRF in young people aged 14 to 17 years compared to usual behavior alone (the control). Secondary aims were to determine the effect on PA levels, enjoyment, psychological need satisfaction, and self-efficacy. Perceptions of usability and acceptability of the apps were also assessed.

## Methods

### Trial Design

A 3-arm parallel RCT was conducted in Auckland, New Zealand. Details on the rationale, design, and methods have been previously described [38]. Ethics approval was obtained from the University of Auckland Human Participants Ethics Committee (10054/2013) and the study was conducted according to the principles of the Declaration of Helsinki. There were no deviations to methods after trial commencement. All participants (and guardians for participants younger than 16 years) provided written informed consent. The trial was registered (Australian New Zealand Clinical Trials Registry: ACTRN12613001030763) in accordance with the requirements of the International Committee of Medical Journal Editors and reported in accordance with the CONSORT statement [39] and the CONSORT-EHEALTH extension [40] (see Multimedia Appendix 1). A brief description of the procedures is provided.

### Participants

Participants were recruited through advertisements in electronic mailing lists, local newspapers, schools, and flyers posted in community locations. Consenting schools and churches allowed the researcher to present a brief outline of the study. Those interested provided contact details and their eligibility was assessed via telephone. If eligible, participant information and informed consent documentation were either mailed or emailed and participants were scheduled to attend a face-to-face baseline assessment at the university.

Eligible participants were aged 14 to 17 years, lived in Auckland, owned an iPod touch or smartphone running at least Android 2.2 or iOS 6.0, and were able to perform physical activities but were not achieving [41] the PA recommendations (ie, at least 60 minutes of MVPA each day). Exclusion criteria were a medical condition limiting ability to exercise safely, previous use of the apps of interest, and inability to comply with the study protocol. Only 1 child per household was eligible to take part.

### Randomization

Participants were enrolled by author AD and were randomly assigned at a 1:1:1 ratio to 1 of 3 conditions. Stratified block randomization in variable blocks was used to maintain balance across gender, an important prognostic factor [2,42]. A biostatistician (YJ) prepared the randomization scheme in advance by using a computer-generated randomization table. Based on the randomization scheme, a research assistant prepared opaque-sealed envelopes containing group referral so that the researcher could not identify group assignment. The envelope was opened by each participant after completion of baseline assessment. Given the nature of the intervention, it was not possible to blind participants. However, allocation concealment was maintained up to the point of randomization.

### Interventions

Commercially available apps targeting fitness were identified during previous work evaluating the most popular (ie, top-20 free and top-20 paid) downloaded apps in the Health and Fitness Category of the iTunes New Zealand store [37]. The control group was compared against 2 other groups, each using an app to promote fitness available on both of the major platforms’ app stores (iTunes and Google Play Store). The 2 intervention groups allowed comparison of an identical delivery approach (ie, stand-alone app), but with distinct design features: (1) use of an immersive app or (2) use of a nonimmersive app. Both apps consisted of a fully automated 8-week training program designed to improve fitness and ability to run 5 km; however, the immersive app featured a game-themed design whereby the training program was embedded with a story where the user is trained to collect supplies and protect a town from zombies. Effective self-regulatory behavior change techniques [43,44] underlie the apps, which allow the users to self-monitor and receive feedback on their training. The apps provided information on running and technique, audio instructions on how to perform the training components, and tracked and displayed progress throughout the program. Other features of the apps include the ability to work out with music on the device’s library and links to associated websites to interact with other users.

Participants randomized to the immersive app group received the Zombies, Run! 5K Training app developed by Six to Start with Naomi Alderman for iOS and the Android platform. It was released worldwide for iOS on October 2012. Even though data on number of downloads is not publicly available, the Google Play Store reports 100,000 to 500,000 installs of this app, and the Zombies, Run! community has more than 800,000 players worldwide [45]. Those randomized to the nonimmersive app group received the Get Running-Couch to 5k app, developed by Splendid Things and Benjohn Barnes for iOS and the Android platform. It was released worldwide for Android on July 2009 and the Google play Store reports 10,000 to 50,000 installs of this app [46]. While the trial was conducted, the apps cost NZ $2.45 to $4.19, depending on the app store. Given the commercialized nature of the apps, updates occurred during the trial; however, no major changes affected their content or the intervention. Videos detailing the features of the apps can be accessed via links on the preceding references.

Following randomization, the respective app was paid for and installed by AD on each participant’s mobile device and a short instruction on the features and settings of the app was given. Participants were encouraged to use their app 3 times per week and work their way through each of the workouts, but because this was a pragmatic study [47], access and usage was allowed to vary (ie, participants were able to use at their own pace, ad libitum, as it would occur on an “everyday life” setting). There were no cointerventions, no supplementary modes of delivery, nor usage of prompts (ie, emails, phone calls, SMS text message) to use the app.

### Control Group

The control group was asked to continue with their usual physical activities for the duration of the study and was not offered any information about increasing PA. Both apps were provided (free of charge) to participants after trial completion.

### Procedure

Assessments were conducted at baseline and 8 weeks at the university by AD. Participants were assessed individually. At both time points, participants completed a field test of CRF (1 mile run/walk test), had their height and weight measured, self-reported their physical activity and related psychological variables, were given an Actigraph accelerometer to wear for the following 7 days (to provide an objective assessment of their free-living PA), and completed a booklet detailing their accelerometer use. AD collected the accelerometers and booklets from the participants’ homes (during the randomization visit that took place after the baseline assessment and at the last visit after the follow-up assessment). Follow-up assessments were not blinded. Participants received a NZ $10 gift card to a local shopping center for each visit to complete study measures (ie, maximum NZ $30 for 3 visits). The vouchers were not conditional on usage of the app; they were offered to compensate for participants’ time and encourage completion of study measures.

### Outcomes

The primary outcome was CRF, assessed with the 1-mile run/walk test. Following the procedures outlined in the Fitnessgram test administration manual, participants were instructed to run and/or walk at their own pace until completing the distance in the shortest possible time [48]. The output, in seconds, was used to estimate VO_2peak_ using a validated prediction equation [49].

Secondary outcomes included anthropometrics, self-reported PA and associated psychological variables, objectively measured PA, and self-reported acceptability and usability of the apps assessed via an exit survey conducted with intervention participants. A series of closed and open-ended questions were asked to determine features perceived as more and less acceptable as well as which features participants found more useful to support their fitness. Body weight (in kg, without shoes) was measured with a Salter scale to 1 decimal place. Height was measured to the nearest 0.1 cm with a Seca stadiometer. Two measurements were taken for each and the means were used for analysis. Body mass index (BMI) was calculated by using the standard equation (weight in kilograms/height in meters squared). BMI-for-age was calculated using the World Health Organization (WHO) growth standards macro [50].

Using instruments validated in this population, participants self-reported (1) physical activity using the Physical Activity Questionnaire for Adolescents (PAQ-A) [51,52], (2) perceived enjoyment using the Physical Activity Enjoyment Scale (PACES) [53,54], (3) perceived competence, autonomy, and relatedness using the Psychological Need Satisfaction in Exercise Scale (PNSES) [55], (4) self-efficacy using the Physical Activity Self-Efficacy Scale (PASES) [56,57], and (5) frequency of app utilization, acceptability, and usability of the app via an exit survey conducted with the participants in the intervention groups.

Participants were instructed to wear the accelerometer (Actigraph GT1M) on their right hip during waking hours for 7 days after each assessment, removing it when engaging in activities involving water and/or contact sports. A 10-second epoch was used and data were aggregated into minute intervals for subsequent processing. To determine valid wear time, periods of more than 60 minutes of consecutive zeroes and days with less than 600 minutes of valid records were removed before data analysis [58]. Participants were included in the analyses if they had 3 or more valid monitoring days [59]. Mean daily time (min) spent in sedentary and light-to-vigorous activities was calculated for each participant using the cut points proposed by Evenson et al to categorize intensities [60,61].

Adverse events were collected at each study visit or voluntarily reported by contacting the researcher. An adverse event was considered serious if it required hospitalization.

### Sample Size Calculation

A total of 51 participants (17 per group) was estimated to provide 80% power and α=.05 overall to detect a difference of 17 seconds in CRF, assuming a 15-second SD in time to complete the 1-mile run/walk test between each of the conditions compared to the control [62].

### Statistical Analyses

Treatment evaluations were performed on the principle of intent-to-treat, including all randomized participants as allocated. Statistical analyses were performed with SAS version 9.4 software (SAS Institute, Cary, NC, USA). All statistical tests were 2-sided at a 5% significance level, with adjustment for multiple comparisons on the primary outcome. Analysis of covariance (ANCOVA) regression model was used to evaluate the main treatment effects on the primary outcome, adjusting for baseline measure and gender. Model-adjusted means, 95% confidence intervals, and *P* values were estimated for each group. Mean differences between groups were tested. A similar approach was used for secondary outcomes. Missing data on the primary outcome were imputed with the baseline value. Per-protocol analyses were conducted on those participants with complete baseline and follow-up data and self-reported adherence to the training program (ie, app used 3 times/week) following the same procedures as in the intention-to-treat analyses.

## Results

### Overview

**Figure 1:**
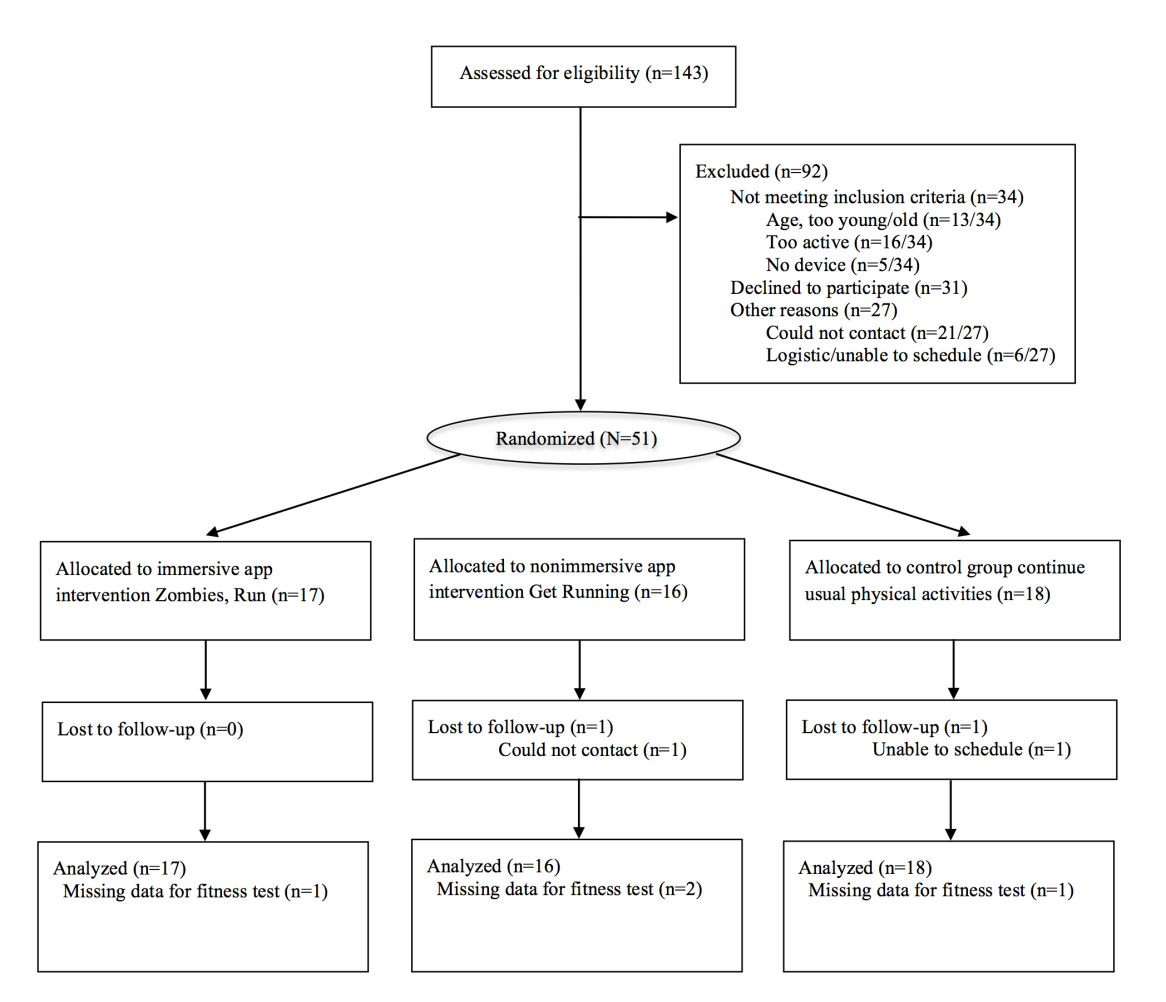
Flow diagram of the Apps for IMproving FITness (AIMFIT) trial. Those who were unable to complete the postintervention fitness assessment due to injury or sickness still completed self-reported outcomes and were included in all analyses.

Recruitment began October 2013 and finished in June 2014. The final follow-up visit was in September 2014. Figure 1 presents the participant flow diagram. Of 143 individuals screened, 51 eligible participants were randomized to the Zombies, Run immersive app intervention (n=17), the Get Running nonimmersive app intervention (n=16), or the control group (n=18). Baseline demographic characteristics of participants are presented in Table 1.

Participants had a mean age of 15.7 years (SD 1.2, range 14-17 years) and a BMI of 22.9 (SD 4.3) kg/m^2^. The majority were NZ European (61%, 31/51), whereas 22% (11/51) were Pacific Islanders, and 57% (29/51) were female. Follow-up assessments at 8 weeks were completed for 17 (100%, 17/17) immersive app group participants, 15 (94%, 15/16) nonimmersive app group participants, and 17 (94%, 17/18) control group participants, which represents an overall retention rate of 96% (49/51) from baseline.

**Table 1:** Baseline demographic and clinical characteristics.

Characteristic		Zombies, Run n=17	Get Running n=16	Control n=18	Total N=51
Age (years), mean (SD)		15.78 (1.11)	15.69 (1.04)	15.55 (1.32)	15.67 (1.15)
**Gender, n (%)**					
	Male	8 (47)	6 (38)	8 (44)	22 (43)
	Female	9 (53)	10 (63)	10 (56)	29 (57)
**Ethnicity, n (%)**					
	Maori	3 (18)	0 (0)	0 (0)	3 (6)
	NZ European	9 (53)	9 (56)	13 (72)	31 (61)
	Pacific	4 (24)	3 (19)	4 (22)	11 (22)
	Asian	0 (0)	3 (19)	1 (6)	4 (8)
	Other	1 (6)	1 (6)	0 (0)	2 (4)
**BMI (kg/m** ^**2**^ **), mean (SD)**		23.17 (3.60)	21.85 (3.14)	23.43 (5.56)	22.85 (4.25)
	BMI-for-age^a^ (z-score), mean (SD)	0.77 (0.86)	0.36 (0.93)	0.64 (1.46)	0.60 (1.12)
**Device, n (%)**					
	iPhone	8 (47)	6 (38)	11 (61)	25 (49)
	Android	5 (29)	7 (44)	5 (28)	17 (33)
	iPod Touch	4 (24)	3 (19)	2 (11)	9 (18)
Estimated VO_2peak_^b^ (mL/kg/min), mean (SD)		43.51 (6.11)	43.58 (5.47)	44.20 (6.95)	43.78 (6.12)

^a^ WHO growth reference.

^b^ Prediction equation from 1-mile run/walk test.

### Primary Outcome

Table 2 lists the effects of the app interventions on the time to complete the 1-mile walk/run fitness test and all secondary outcomes at 8 weeks. On average, time to complete the fitness test decreased in both app groups, but there were no statistically significant differences observed between the intervention groups and the control (immersive app group: adjusted mean difference –28.4 sec, 95% CI –66.5 to 9.8, *P=*.20; nonimmersive app group: adjusted mean difference –24.7 sec, 95% CI –63.5 to 14.2 sec, *P=*.32). To facilitate interpretation of test scores, estimated VO_2 peak_ [49] at 8 weeks was 44.09, 44.59, and 43.44 mL/kg/min for the immersive app, nonimmersive app, and control groups, respectively.

**Table 2:** Treatment effects at 8 weeks.

Outcome		Zombies, Run (1), mean (SD)		Get Running (2), mean (SD)		Control (3), mean (SD)		Adjusted difference^a^ (95% CI) at 8 weeks			
		Baseline	8 week	Baseline	8 week	Baseline	8 week	1 vs 3	*P*	2 vs 3	*P*
Time to complete 1-mile walk/run (sec)		574.06 (145.68)	560.06 (139.27)	586.56 (129.74)	576.75 (147.91)	585.89 (600.17)	600.17 (191.38)	–28.36 (–66.54, 9.82)	.20	–24.67 (–63.51, 14.18)	.32
PAQ-A		2.20 (0.66)	2.27 (0.53)	2.09 (0.73)	2.31 (0.74)	2.30 (0.67)	2.21 (0.62)	0.14 (–0.26, 0.54	.78	0.23 (–0.18, 0.64)	.42
PACES		4.08 (0.47)	4.00 (0.46)	3.99 (0.46)	3.85 (0.46)	3.96 (0.58)	4.00 (0.57)	–0.10 (–0.33, 0.13)	.62	–0.17 (–0.40, 0.06)	.19
**PNSES**		4.52 (0.69)	4.49 (0.78)	4.48 (0.89)	4.56 (0.56)	4.67 (0.85)	4.68 (0.76)	–0.08 (–0.46, 0.31)	.95	0.01 (–0.38, 0.40)	>.99
	Competence	4.27 (0.83)	4.24 (0.94)	4.25 (1.09)	4.32 (0.94)	4.54 (1.17)	4.52 (1.22)	–0.08 (–0.67, 0.51)	.98	0.03 (–0.57, 0.63)	.99
	Autonomy	4.87 (0.98)	4.92 (0.94)	4.94 (0.78)	5.21 (0.56)	4.94 (0.99)	4.84 (0.91)	0.12 (–0.44, 0.69)	.93	0.36 (–0.22, 0.94)	.34
	Relatedness	4.40 (0.79)	4.31 (0.87)	4.26 (1.15)	4.14 (0.91)	4.53 (1.16)	4.67 (1.27)	–0.26 (–0.88, 0.35)	.65	–0.34 (–0.97, 0.29)	.45
PASES		2.50 (0.32)	2.44 (0.40)	2.38 (0.47)	2.43 (0.31)	2.38 (0.41)	2.39 (0.34)	–0.02 (–0.24, 0.19)	.99	0.04 (–0.18, 0.26)	.96
Average daily valid use (min)		801.16 (87.85)	784.43 (93.69)	819.39 (85.45)	831.08 (78.74)	815.59 (57.14)	814.12 (73.61)	–21.72 (–84.00, 40.56)	.77	13.71 (–49.56, 76.99)	.93
Average daily activity counts (counts/min)		354.27 (123.75)	341.21 (146.22)	269.97 (82.72)	270.20 (84.77)	364.83 (153.81)	327.53 (140.02)	17.74 (–63.07, 98.55)	.93	0.90 (–85.41, 87.22)	>.99
Average daily time spent in sedentary activities (min)		526.63 (106.37)	535.22 (113.90)	553.64 (96.80)	570.63 (95.58)	529.43 (94.64)	548.18 (94.11)	–10.94 (–69.83, 48.00)	.96	3.95 (–56.26, 64.16)	.99
Average daily time spent in light PA (min)		237.41 (73.32)	216.17 (67.46)	244.46 (61.97)	237.11 (58.45)	250.39 (64.05)	235.40 (63.89)	–10.54 (–53.96, 32.88)	.91	4.12 (–39.94, 48.17)	.99
Average daily time spent in moderate PA (min)		30.28 (14.28)	25.07 (12.83)	17.03 (7.81)	18.03 (10.20)	25.52 (13.75)	22.33 (11.69)	1.42 (–7.96, 10.81)	.98	–1.71 (–11.51, 8.10)	.96
Average daily time spent in vigorous PA (min)		6.84 (6.03)	7.97 (9.16)	4.26 (4.21)	5.30 (4.34)	10.26 (11.72)	8.22 (9.22)	1.26 (–3.82, 6.33)	.90	0.52 (–4.79, 5.83)	.99
Average daily time spent in MVPA (min)		37.12 (16.84)	33.04 (20.61)	21.29 (11.25)	23.34 (14.04)	35.78 (22.54)	30.54 (17.99)	1.74 (–11.45, 14.93)	.98	–1.82 (–16.00, 12.36)	.99

^a^ Adjusted for baseline, gender, and multiple comparisons.

### Secondary Outcomes

No intervention effects were found for self-reported secondary outcomes of physical activity (PAQ-A; immersive app group: adjusted mean difference 0.14, 95% CI –0.26 to 0.54, *P=*.78; nonimmersive app group: adjusted mean difference 0.23, 95% CI –0.18 to 0.64, *P*=.42) or its predictors of perceived enjoyment (PACES), perceived competence, autonomy, and relatedness (PNSES), or self-efficacy (PASES) (see Table 2 for all outcomes).

For accelerometry, 48 of 51 (94%) participants provided valid data for analysis at baseline, whereas compliance with wearing the device slightly decreased at postintervention (46/51, 90%). Group assignment did not have a significant effect on overall activity (ie, mean counts per minute) or mean daily time spent in MVPA. Compared to the control group, mean baseline daily time spent in MVPA-, gender-, and multiple comparisons-adjusted time in MVPA difference was 1.74 min (95% CI –11.45 to 14.93, *P=*.98) and –1.82 min (95% CI –16.00 to 12.36, *P*=.99) for the immersive and nonimmersive app groups, respectively.

A total of 6 adverse events (1 serious) were reported in 6 participants, 4 of which were in the control group (ankle injury-2 events, lower back pain, and hospitalization because of tonsils removal) and 1 in each of the intervention groups (ankle injury-2 events). None of the adverse events were deemed related to the study intervention.

Approximately two-thirds of participants in the intervention groups reported using the app either 2 (10/32, 31%) or 3 times per week (10/32, 31%), whereas 8 of 32 (25%) only used it 1 time per week (see Multimedia Appendix 2). No differences were evident on timing of use (ie, weekday, weekend, morning, afternoon, evening). Apps were mostly used outdoors (eg, street, park vs gym, home treadmill) and while alone (n=13 alone, n=7 with friend, and n=9 with family).

For the app Zombies, Run!, the features mostly used by participants were the “workout mission tasks” (n=14) and “story and run log” of completed workouts (n=10), whereas social networking features (“share my runs”: n=0; “ZombieLink account”: n=3) were seldom or never used. Results were similar when participants reported the features they liked (“workout mission tasks”: n=14; “story and run log”: n=8) and disliked (“share my runs”: n=5; “ZombieLink account”: n=5).

For the app Get Running, the feature mostly used by participants was the description of the “week-runs” (n=13), whereas only 1 participant reported using the social networking feature “status updates.” The description of the “week-runs” was also the feature participants predominantly liked (n=11), whereas the main feature disliked was the “status updates” (n=3).

Regardless of the app used, similar themes emerged when participants reported their willingness and motives to continue using their app after study participation. Those willing to continue stated personal benefits (eg, “It will help me to build my fitness”, “Because I can improve how far I run”) and app-related motives (eg, “A fun way to get fit”, “Because it is an enjoyable alternative to exercise”). For those unwilling to continue, “not enough time” was the most common barrier, followed by lack of interest (eg, “I didn’t find the app engaging enough”). The nonimmersive app received less positive feedback around motivational aspects (eg, “Using the app became too tedious”).

Overall, participants perceived the layout of the apps and menus as well structured and “straightforward” to use. Being able to receive clear instructions (eg, “Tells me what to do and when”), listen to their own music during the training sessions, task difficulty increasing gradually, and encouragement provided were features highlighted as useful to support participants’ fitness. Some also considered it helpful if the app allowed choosing between different goals and activities (eg, “I prefer to run to my own goals”). For the immersive app, the storyline (“The back story made it interesting”) and the ability to track progress (ie, app used the device’s Global Positioning System [GPS] and/or accelerometer to log distance) (eg, “It records the distance you ran and your time so you are able to view it for next time and compare”) were also reported as important features.

The majority of participants (21/32, 66%) had no prior experience of using their smartphone for PA purposes. Examples of prior experience included listening to music while engaging in PA or previous use of free apps (eg, MyFitnessPal). Overall, 81% (26/32) were interested in trying different PA-promoting apps in the future.

In prespecified per-protocol analyses (ie, the app was used 3 times/week), there were statistically significant differences observed on the primary outcome between the nonimmersive app group and the control (adjusted mean difference –79.39 sec, 95% CI –133.01 to –25.77, *P*=.003). Time to complete the fitness test also decreased for the immersive app group compared to the control, with the difference not meeting statistical significance (adjusted mean difference –55.29 sec, 95% CI –111.46 to 0.88, *P*=.06). No intervention effects were found for self-reported secondary outcomes of PA, its predictors, or accelerometry.

## Discussion

### Principal Findings

This is the first randomized trial comparing the effects of a stand-alone immersive mobile app and a nonimmersive app on CRF, PA levels, and its predictors in young people. Key findings were that fitness improved in both app groups, but these did not significantly differ from the control. Despite the availability of readily available commercial apps to improve health behavior, these findings suggest that, compared to usual care, no major improvements were found for these 2 top downloaded apps.

The small increases in fitness in the present trial (0.6 to 1.0 mL/kg/min) were lower than those observed in a Cochrane review of school-based PA interventions, which found increases of 1.6 to 3.7 mL/kg/min in VO_2peak_ [14]. Similarly, Baquet et al [63], who reviewed the effects of training programs in young people’s aerobic fitness, showed improvements on average of 5% to 6% in VO_2peak,_ whereas fitness only improved 1.3% to 2.3% in this study. The training programs of the apps were consistent with the frequency and duration of programs shown to improve VO_2peak_ [63] (ie, incorporating 3 sessions per week, each lasting 30 minutes to 1 hour). However, use of the apps during the AIMFIT study was ad libitum and not closely monitored, which is how app use would likely occur in real life. Smaller fitness improvements in the current study can most likely be attributed to lower adherence to app use (only 31.3% reported using 3 times/week) or due to participants not having exercised at a sufficient intensity to produce larger fitness effects [63], as observed by no changes in accelerometry-measured MVPA.

Although apps have the potential to increase the reach of health behavior change interventions, our results mirror recent research highlighting that only some participants will consistently use an offered app (approximately 20%) [64] or sustain use over time (approximately 3%) [65]. App design, features, and usability are important factors that impact users’ engagement with an app. Although in AIMFIT both app groups produced comparable fitness effects, the design and features of the immersive app received more positive feedback (and no dropout) and, therefore, these aspects should be considered for future app development. Although typical app use is intermittent [65] and unlikely to induce the more intensive type of immersion observed in other videogame play, incorporating gaming elements that entangle the virtual world (ie, leisure) with actual experience (ie, exertion) could potentially increase user engagement and sustained use.

An important consideration of app content is whether or not they incorporate behavior change techniques (BCTs). Further, modeling, providing consequences for behavior, providing information on others’ approval, prompting intention formation, self-monitoring, and a behavioral contract were identified as effective BCTs for increasing PA in young people in a recent meta-regression [66]. A notable exception is providing instruction, which was associated with decreased effectiveness of PA interventions in young people, but has been shown to be highly present in top-ranked PA apps [37], including the ones hereby investigated. Providing instructions and not incorporating other effective BCTs or incorrectly applying BCTs (ie, not considering the parameters for them to be effective) may have undermined app efficacy.

Consistent with the primary outcome findings, we found no changes in any of the measured psychological variables. Fulfillment of the 3 basic psychological needs (ie, autonomy, competence, and relatedness)—key elements in the development of intrinsic motivation required to drive behavioral change [67]—did not change. This could be explained by the nature of the training programs, which were based on provision of instructions and may have been perceived as too prescriptive and controlling. Perhaps the alternatives provided by the apps (ie, walking, jogging, sprinting, running) lacked choice and encouragement of the participants’ initiative and, therefore, did not support their autonomy. In addition, skill acquisition and feelings of competence did not change as a function of the intervention. This suggests the BCTs in the apps were insufficient to manipulate these variables or that app use was not intensive enough to evoke change. Further, the apps incorporate features that could capitalize on relatedness and social support, such as online communities, but some of these features load outside of the apps (eg, ZombieLink), which may have contributed to poor use of such features. Interestingly, enjoyment, which is associated with adherence to PA programs, did not change.

### Strengths and Limitations

A major strength of AIMFIT was the use of a RCT design to determine the effectiveness of 2 off-the-shelf commercially available interventions. We chose a pragmatic approach in which participants used their own device and apps were used ad libitum. Contact with participants was minimal, which reflects app use in a real-world context and therefore increases the generalizability of the findings. Moreover, the primary outcome was assessed objectively with a valid and reliable measurement, as well as PA via accelerometry, which adds to the study’s internal validity. Unlike the commonly observed high attrition in eHealth and mHealth interventions, follow-up assessments were completed for 96% of participants, which represents a high retention rate. Further, using paid apps instead of free ones has likely reduced the chances of contamination and/or cointerventions. Although the accessibility of these apps on the app stores could have led participants to download them, our study sample was a young population and the apps under investigation required purchase. Although inexpensive, purchasing an app on an app store requires a credit card, which young people do not typically own.

The major limitation of this study was its low statistical power and small sample size. We based our sample size calculation on a smaller standard deviation of the primary outcome than the actual standard deviation observed, which meant the power of the trial was smaller than 80%. We used readily available apps and consequently were limited to the decisions made by the app developers on content, duration of the program, and design features. This also meant that we were unable to access data on app utilization (eg, menus accessed in the app). Further, the relatively short duration of the programs precluded investigation of long-term effects or sustainability. This study also highlights that the peer-reviewed literature will always lag behind consumer technology life cycles because during the lifetime of this study innovative apps were developed at a rate that far outpaced our capacity to test them.

### Implications

Among app users, fitness apps are the most popular (78% users in 2014 compared to 39% in 2013) [35]. However, off-the-shelf common commercial mobile apps used as a stand-alone approach to change fitness and promote PA in insufficiently active individuals do not appear effective. Future use as part of a multifaceted approach to increase fitness and to promote PA may be useful. The current one-size-fits-all approach of most common commercial mobile apps is limiting for both users and researchers. More tailored approaches, which are dynamic and responsive to changes in PA behavior over time, are required to improve the ease of use of apps, user engagement, and the apps’ sustained use.

### Conclusions

Readily available commercial apps as a stand-alone intervention to improve fitness and increase PA in young people did not increase CRF compared to usual care. Given that smartphone technology appears to resonate with young people and that this type of self-guided intervention has the potential to increase reach at a low cost, this may be best suited as part of a multicomponent intervention, providing additional support and encouragement to the participants (eg, maintenance phases).

## Appendix

Multimedia Appendix 1 CONSORT-EHEALTH checklist V1.6.2 [40].

Multimedia Appendix 2 Supplementary word file containing graphics with exit survey questions for intervention participants.

## Abbreviations

BCT: behavior change technique
BMI: body mass index
CRF: cardiorespiratory fitness
MVPA: moderate-to-vigorous physical activity
PA: physical activity
PACES: Physical Activity Enjoyment Scale
PAQ-A: Physical Activity Questionnaire for Adolescents
PASES: Physical Activity Self-Efficacy Scale
PNSES: Psychological Need Satisfaction in Exercise Scale
RCT: randomized controlled trial
SMS: short message service

## References

[1] Hallal PC, Andersen LB, Bull FC, Guthold R, Haskell W, Ekelund U, Lancet Physical Activity Series Working Group (2012). Global physical activity levels: surveillance progress, pitfalls, and prospects. Lancet.

[2] Clinical Trials Research Unit, Synovate (2010). A National Survey of Children and Young People’s Physical Activity and Dietary Behaviours in New Zealand: 2008/09: Key Findings.

[3] Tomkinson GR, Annandale M, Ferrar K (2013). Global changes in cardiovascular endurance of children and youth since 1964: systematic analysis of 25 million fitness test results from 28 countries. Circulation.

[4] Lee I, Shiroma EJ, Lobelo F, Puska P, Blair SN, Katzmarzyk PT, Lancet Physical Activity Series Working Group (2012). Effect of physical inactivity on major non-communicable diseases worldwide: an analysis of burden of disease and life expectancy. Lancet.

[5] Ekelund U, Luan J, Sherar LB, Esliger DW, Griew P, Cooper A, International Children's Accelerometry Database (ICAD) Collaborators (2012). Moderate to vigorous physical activity and sedentary time and cardiometabolic risk factors in children and adolescents. JAMA.

[6] Kaminsky LA, Arena R, Beckie TM, Brubaker PH, Church TS, Forman DE, Franklin BA, Gulati M, Lavie CJ, Myers J, Patel MJ, Piña IL, Weintraub WS, Williams MA, American Heart Association Advocacy Coordinating Committee‚ Council on Clinical Cardiology‚Council on Nutrition‚ Physical ActivityMetabolism (2013). The importance of cardiorespiratory fitness in the United States: the need for a national registry: a policy statement from the American Heart Association. Circulation.

[7] Ortega FB, Ruiz JR, Castillo MJ, Sjöström M (2008). Physical fitness in childhood and adolescence: a powerful marker of health. Int J Obes (Lond).

[8] Shuval K, Finley CE, Barlow CE, Gabriel KP, Leonard D, Kohl HW (2014). Sedentary behavior, cardiorespiratory fitness, physical activity, and cardiometabolic risk in men: the cooper center longitudinal study. Mayo Clin Proc.

[9] Kodama S, Saito K, Tanaka S, Maki M, Yachi Y, Asumi M, Sugawara A, Totsuka K, Shimano H, Ohashi Y, Yamada N, Sone H (2009). Cardiorespiratory fitness as a quantitative predictor of all-cause mortality and cardiovascular events in healthy men and women: a meta-analysis. JAMA.

[10] Marques A, Santos R, Ekelund U, Sardinha LB (2015). Association between physical activity, sedentary time, and healthy fitness in youth. Med Sci Sports Exerc.

[11] Dumith SC, Gigante DP, Domingues MR, Kohl HW (2011). Physical activity change during adolescence: a systematic review and a pooled analysis. Int J Epidemiol.

[12] Corder K, Sharp SJ, Atkin AJ, Griffin SJ, Jones AP, Ekelund U, van Sluijs EM (2014). Change in objectively measured physical activity during the transition to adolescence. Br J Sports Med.

[13] Telama R (2009). Tracking of physical activity from childhood to adulthood: a review. Obes Facts.

[14] Dobbins M, Husson H, DeCorby K, LaRocca RL (2013). School-based physical activity programs for promoting physical activity and fitness in children and adolescents aged 6 to 18. Cochrane Database Syst Rev.

[15] Taber DR, Stevens J, Murray DM, Elder JP, Webber LS, Jobe JB, Lytle LA (2009). The effect of a physical activity intervention on bias in self-reported activity. Ann Epidemiol.

[16] van Sluijs EM, McMinn AM, Griffin SJ (2007). Effectiveness of interventions to promote physical activity in children and adolescents: systematic review of controlled trials. BMJ.

[17] Metcalf B, Henley W, Wilkin T (2012). Effectiveness of intervention on physical activity of children: systematic review and meta-analysis of controlled trials with objectively measured outcomes (EarlyBird 54). BMJ.

[18] Hallal P, Bauman A, Heath G, Kohl H, Lee I, Pratt M (2012). Physical activity: more of the same is not enough. The Lancet.

[19] Kontos E, Blake KD, Chou WS, Prestin A (2014). Predictors of eHealth usage: insights on the digital divide from the Health Information National Trends Survey 2012. J Med Internet Res.

[20] Eshet-Alkalai Y, Chajut E (2009). Changes over time in digital literacy. Cyberpsychol Behav.

[21] Madden M, Lenhart A, Duggan M, Cortesi S, Gasser U (2013). Teens and Technology 2013.

[22] Madden M, Lenhart A, Cortesi S, Gasser U (2013). Teens and Mobile Apps Privacy.

[23] Crothers C, Gibson A, Smith P, Bell A, Miller M (2014). Internet Trends in New Zealand 2007–2013.

[24] Crothers C, Gibson A, Smith P, Bell A, Miller M (2013). The Internet in New Zealand 2013.

[25] Reseach2Guidance (2014). Mobile Health Economics.

[26] Kuehn BM (2015). Is there an app to solve app overload?. JAMA.

[27] Tate EB, Spruijt-Metz D, O'Reilly G, Jordan-Marsh M, Gotsis M, Pentz MA, Dunton GF (2013). mHealth approaches to child obesity prevention: successes, unique challenges, and next directions. Transl Behav Med.

[28] Hurling R, Catt M, Boni M, Fairley B, Hurst T, Murray P, Richardson A, Sodhi J (2007). Using internet and mobile phone technology to deliver an automated physical activity program: randomized controlled trial. J Med Internet Res.

[29] Fjeldsoe BS, Miller YD, Marshall AL (2010). MobileMums: a randomized controlled trial of an SMS-based physical activity intervention. Ann Behav Med.

[30] Conroy MB, Yang K, Elci OU, Gabriel KP, Styn MA, Wang J, Kriska AM, Sereika SM, Burke LE (2011). Physical activity self-monitoring and weight loss: 6-month results of the SMART trial. Med Sci Sports Exerc.

[31] Kirwan M, Duncan MJ, Vandelanotte C, Mummery WK (2012). Using smartphone technology to monitor physical activity in the 10,000 Steps program: a matched case-control trial. J Med Internet Res.

[32] Nollen NL, Mayo MS, Carlson SE, Rapoff MA, Goggin KJ, Ellerbeck EF (2014). Mobile technology for obesity prevention: a randomized pilot study in racial- and ethnic-minority girls. Am J Prev Med.

[33] Blackman KC, Zoellner J, Berrey LM, Alexander R, Fanning J, Hill JL, Estabrooks PA (2013). Assessing the internal and external validity of mobile health physical activity promotion interventions: a systematic literature review using the RE-AIM framework. J Med Internet Res.

[34] Fanning J, Mullen SP, McAuley E (2012). Increasing physical activity with mobile devices: a meta-analysis. J Med Internet Res.

[35] Citrix ByteMobile (2015). Mobile Analytics Report.

[36] Fiordelli M, Diviani N, Schulz PJ (2013). Mapping mHealth research: a decade of evolution. J Med Internet Res.

[37] Direito A, Dale LP, Shields E, Dobson R, Whittaker R, Maddison R (2014). Do physical activity and dietary smartphone applications incorporate evidence-based behaviour change techniques?. BMC Public Health.

[38] Direito A, Jiang Y, Whittaker R, Maddison R (2015). Smartphone apps to improve fitness and increase physical activity among young people: protocol of the Apps for IMproving FITness (AIMFIT) randomized controlled trial. BMC Public Health.

[39] Schulz KF, Altman DG, Moher D (2010). CONSORT 2010 statement: updated guidelines for reporting parallel group randomized trials. Ann Intern Med.

[40] Eysenbach G (2011). CONSORT-EHEALTH: improving and standardizing evaluation reports of Web-based and mobile health interventions. J Med Internet Res.

[41] Prochaska JJ, Sallis JF, Long B (2001). A physical activity screening measure for use with adolescents in primary care. Arch Pediatr Adolesc Med.

[42] Nader PR, Bradley RH, Houts RM, McRitchie SL, O'Brien M (2008). Moderate-to-vigorous physical activity from ages 9 to 15 years. JAMA.

[43] Webb TL, Joseph J, Yardley L, Michie S (2010). Using the internet to promote health behavior change: a systematic review and meta-analysis of the impact of theoretical basis, use of behavior change techniques, and mode of delivery on efficacy. J Med Internet Res.

[44] Michie S, Abraham C, Whittington C, McAteer J, Gupta S (2009). Effective techniques in healthy eating and physical activity interventions: a meta-regression. Health Psychol.

[45] Six to Start.

[46] Splendid Things.

[47] Macpherson H (2004). Pragmatic clinical trials. Complement Ther Med.

[48] Meredith M, Welk G, The Cooper Institute (2013). Fitnessgram & Activitygram Test Administration Manual-Updated 4th Edition.

[49] Cureton KJ, Sloniger MA, O'Bannon JP, Black DM, McCormack WP (1995). A generalized equation for prediction of VO2peak from 1-mile run/walk performance. Med Sci Sports Exerc.

[50] de Onis M, Onyango AW, Borghi E, Siyam A, Nishida C, Siekmann J (2007). Development of a WHO growth reference for school-aged children and adolescents. Bull World Health Organ.

[51] Janz KF, Lutuchy EM, Wenthe P, Levy SM (2008). Measuring activity in children and adolescents using self-report: PAQ-C and PAQ-A. Med Sci Sports Exerc.

[52] Kowalski K, Crocker P, Kowalski N (1997). Convergent validity of the physical activity questionnaire for adolescents. Pediatric Exercise Science.

[53] Dunton GF, Tscherne J, Rodriguez D (2009). Factorial validity and gender invariance of the physical activity enjoyment scale (PACES) in older adolescents. Res Q Exerc Sport.

[54] Motl RW, Dishman RK, Saunders R, Dowda M, Felton G, Pate RR (2001). Measuring enjoyment of physical activity in adolescent girls. Am J Prev Med.

[55] Wilson P, Rogers W, Rodgers W, Wild T (2006). The psychological need satisfaction in exercise scale. Journal of Sport & Exercise Psychology.

[56] Motl RW, Dishman RK, Trost SG, Saunders RP, Dowda M, Felton G, Ward DS, Pate RR (2000). Factorial validity and invariance of questionnaires measuring social-cognitive determinants of physical activity among adolescent girls. Prev Med.

[57] Bartholomew J, Loukas A, Jowers E, Allua S (2006). Validation of the physical activity self-efficacy scale: testing measurement invariance between Hispanic and Caucasian children. J Phys Act Health.

[58] Mâsse LC, Fuemmeler BF, Anderson CB, Matthews CE, Trost SG, Catellier DJ, Treuth M (2005). Accelerometer data reduction: a comparison of four reduction algorithms on select outcome variables. Med Sci Sports Exerc.

[59] Basterfield L, Adamson AJ, Pearce MS, Reilly JJ (2011). Stability of habitual physical activity and sedentary behavior monitoring by accelerometry in 6- to 8-year-olds. J Phys Act Health.

[60] Trost SG, Loprinzi PD, Moore R, Pfeiffer KA (2011). Comparison of accelerometer cut points for predicting activity intensity in youth. Med Sci Sports Exerc.

[61] Evenson KR, Catellier DJ, Gill K, Ondrak KS, McMurray RG (2008). Calibration of two objective measures of physical activity for children. J Sports Sci.

[62] (2013). The President’s Challenge Program.

[63] Baquet G, van Praagh E, Berthoin S (2003). Endurance training and aerobic fitness in young people. Sports Med.

[64] Goh G, Tan NC, Malhotra R, Padmanabhan U, Barbier S, Allen JC, Østbye T (2015). Short-term trajectories of use of a caloric-monitoring mobile phone app among patients with type 2 diabetes mellitus in a primary care setting. J Med Internet Res.

[65] Helander E, Kaipainen K, Korhonen I, Wansink B (2014). Factors related to sustained use of a free mobile app for dietary self-monitoring with photography and peer feedback: retrospective cohort study. J Med Internet Res.

[66] Brannon EE, Cushing CC (2015). A systematic review: is there an app for that? Translational science of pediatric behavior change for physical activity and dietary interventions. J Pediatr Psychol.

[67] Ryan R, Frederick C, Lepes D, Rubio N, Sheldon K (1997). Intrinsic motivation and exercise adherence. Int J Sport Psychol.

