# Supplementary material for: Apps for IMproving FITness and Increasing Physical Activity Among Young People: The AIMFIT Pragmatic Randomized Controlled Trial
Source: J Med Internet Res. 2015 Aug 27;17(8):e210. doi: 10.2196/jmir.4568 (PMC4642788; doi:10.2196/jmir.4568)
Supplement: Multimedia Appendix 1 [file jmir_v17i8e210_app1.pdf]

CONSORT-EHEALTH Checklist V1.6.2 Report

(based on CONSORT-EHEALTH V1.6), available at [http://tinyurl.com/consort-ehealth-v1-6].

4568

Date completed

4/21/2015 2:21:35

by

Artur Direito

Apps for IMproving FITness and increase physical activity among young people: The AIMFIT pragmatic randomized controlled trial

TITLE

1a-i) Identify the mode of delivery in the title

"Apps for"...

1a-ii) Non-web-based components or important co-interventions in title

N/A, No co-interventions, the apps are used as a stand-alone intervention tool

1a-iii) Primary condition or target group in the title

"among young people"
"improving fitness and increase physical activity"

ABSTRACT

1b-i) Key features/functionalities/components of the intervention and comparator in the METHODS section of the ABSTRACT

..."allocated at random to one of three conditions: 1) use of an immersive app, 2) use of a non-immersive app, or 3) usual behavior (control). Both smartphone apps consisted of a fully automated eight-week training program designed to improve fitness and ability to run 5 km; however, the immersive app featured a game-themed design and narrative".

1b-ii) Level of human involvement in the METHODS section of the ABSTRACT

"Both smartphone apps consisted of a fully automated eight-week training program designed to"...

1b-iii) Open vs. closed, web-based (self-assessment) vs. face-to-face assessments in the METHODS section of the ABSTRACT

"Participants were recruited through advertisements in electronic mailing lists, local newspapers, flyers posted in community locations, and presentations at schools."
"...using data collected face-to-face at baseline and 8 weeks".
"...The primary outcome was cardiorespiratory fitness, objectively assessed as time to complete the one mile run/walk test at 8 weeks. Secondary outcomes were physical activity levels (accelerometry and self-reported)".

1b-iv) RESULTS section in abstract must contain use data

"Fifty-one participants were randomized to the immersive app intervention (n=17), non-immersive app intervention (n=16), or the control (n=18)"
"Overall retention rate was 96%. There was no significant intervention effect on the primary outcome using either of the apps. Compared to the control, time to complete the fitness test was -28.4 sec shorter (95% CI -66.5 to 9.82 sec, P = .20) for the immersive app group and -24.7 sec (95% CI -63.5 to 14.2 sec, P = .32) for the non-immersive app group. No significant intervention effects were found for secondary outcomes"

1b-v) CONCLUSIONS/DISCUSSION in abstract for negative trials

"While apps have the ability to increase reach at a low cost, our pragmatic approach using commercially, readily available apps as a stand-alone instrument did not have a significant effect on fitness."
We highlight that the negative results are attributable to insufficient use of the apps in the discussion section.

INTRODUCTION

2a-i) Problem and the type of system/solution

"Worldwide, 80.3% (95% CI 80.1–80.5) of adolescents aged 13-15 years do not achieve current physical activity (PA) recommendations"
"Insufficient activity is associated with increased risk of chronic diseases"
"Many existing interventions, including school-based interventions, are limited because they are resource intensive"
"Given young people's ubiquitous use of mobile phones and increased digital literacy, interventions leveraging this technology may provide a promising intervention for this population."
"This high engagement with mobile technology offers an ideal opportunity to leverage the benefits of mobile interventions for health (mHealth), including lower participant burden and flexibility"

2a-ii) Scientific background, rationale: What is known about the (type of) system

"The effectiveness of mHealth delivered interventions to promote physical activity that use texting or short message service (SMS), PDAs, and apps in addition to other components has been examined. Unlike SMS, which has a substantial body of literature supporting its use, more complex interventions that capitalize on the computational power of smartphones have only recently been developed. Particularly, few studies have used an entirely mHealth device-based approach to deliver health behaviour change interventions."
"Thus, at present, there is a lack of scientific evidence assessing publicly available apps to promote physical activity. Because these apps are commercially available to the public on app stores, it is important to determine whether they are effective"

METHODS

3a) CONSORT: Description of trial design (such as parallel, factorial) including allocation ratio

"the primary aim of the Apps for IMproving FITness (AIMFIT) trial was to evaluate the effectiveness of two popular commercially available smartphone apps for improving cardiorespiratory fitness in young people aged 14 to 17 years, compared to usual behavior alone (the control). Secondary aims were to determine the effect on physical activity levels, enjoyment, psychological need satisfaction, and self-efficacy. Perceptions of usability and acceptability of the apps were also assessed."

3b) CONSORT: Important changes to methods after trial commencement (such as eligibility criteria), with reasons

"There were no deviations to methods after trial commencement".

3b-i) Bug fixes, Downtimes, Content Changes

"Given the commercialized nature of the apps, updates occurred during the trial; however, no major changes affected their content or the intervention"

4a) CONSORT: Eligibility criteria for participants

"Eligible participants were aged 14-17 years, lived in Auckland, owned an iPod touch or smartphone running at least Android 2.2 or iOS 6.0, and were able to perform physical activities but were not achieving [34] the PA recommendations (i.e. at least 60 minutes of moderate to vigorous PA each day). Exclusion criteria were a medical condition limiting ability to exercise safely, previous use of the apps of interest, and inability to comply with the study protocol. Only one child per household was eligible to take part."

4a-i) Computer / Internet literacy

"Eligible participants (...) owned an iPod touch or smartphone running at least Android 2.2 or iOS 6.0".
"Exclusion criteria were (...) inability to comply with the study protocol".
We only included participants that owned and used an eligible device. Moreover, during the phone call to determine eligibility, we confirmed the participants' digital/computer literacy by having the participant checking their device's operative system.

4a-ii) Open vs. closed, web-based vs. face-to-face assessments:

"Participants were recruited through advertisements in electronic mailing lists, local newspapers, and flyers posted in community locations. Consenting schools and churches allowed the researcher to present a brief outline of the study. Those interested provided contact details and their eligibility was assessed via telephone. If eligible, participant information and informed consent documentation were posted and participants were scheduled to attend a baseline assessment at the University."
Our primary outcome (objectively assessed time to complete the one mile run/walk) required a face-to-face assessment.

4a-iii) Information giving during recruitment
"Consenting schools and churches allowed the researcher to present a brief outline of the study. Those interested provided contact details and their eligibility was assessed via telephone. If eligible, participant information and informed consent documentation were posted and participants were scheduled to attend a face-to-face baseline assessment at the University"

4b) CONSORT: Settings and locations where the data were collected
"Assessments were conducted at baseline and 8-weeks at the University by AD. Participants were assessed individually."

4b-i) Report if outcomes were (self-)assessed through online questionnaires
N/A
"Assessments were conducted at baseline and 8-weeks at the University by AD. Participants were assessed individually. At each time point, subjects completed a field test of cardiorespiratory fitness (one mile run/walk test), had their height and weight measured, self-reported their physical activity and related psychological variables, were given an Actigraph accelerometer to wear for the following seven days (to provide an objective assessment of their free-living PA), and completed a booklet detailing their accelerometer use".

4b-ii) Report how institutional affiliations are displayed
N/A
The study advertisements were reviewed and were in accordance with the guidelines of the University of Auckland Human Participants Ethics Committee.

5) CONSORT: Describe the interventions for each group with sufficient details to allow replication, including how and when they were actually administered

5-i) Mention names, credential, affiliations of the developers, sponsors, and owners
"Participants randomized to the immersive app group received the “Zombies, Run! 5K Training” app, developed by Six to Start with Naomi Alderman for iOS and the Android platform. It was released worldwide for iOS on October 2012. Those randomized to the non-immersive app group received the “Get Running – Couch to 5k” app, developed by Splendid Things and Benjohn Barnes for iOS and the Android platform. It was released worldwide for Android on July 2009"

5-ii) Describe the history/development process
"Participants randomized to the immersive app group received the “Zombies, Run! 5K Training” app, developed by Six to Start with Naomi Alderman for iOS and the Android platform. It was released worldwide for iOS on October 2012. Those randomized to the non-immersive app group received the “Get Running – Couch to 5k” app, developed by Splendid Things and Benjohn Barnes for iOS and the Android platform. It was released worldwide for Android on July 2009".
Further details available on the references included to the developers' websites.

5-iii) Revisions and updating
"Given the commercialized nature of the apps, updates occurred during the trial; however, no major changes affected their content or the intervention."

5-iv) Quality assurance methods
N/A

5-v) Ensure replicability by publishing the source code, and/or providing screenshots/screen-capture video, and/or providing flowcharts of the algorithms used

N/A
The references in the manuscript provide links to screenshots and videos of the apps.
"Videos detailing the features of the apps can be accessed via links on the above references."

5-vi) Digital preservation
The links to screenshots and videos of the apps will be archived using webcitation.org.

5-vii) Access
"Following randomization, the respective app was paid for and installed by AD on each participant’s mobile device and a short instruction on the features and settings of the app was given".
"Participants were encouraged to use their app three times per week and work their way through each of the workouts, but since this was a pragmatic study, access and usage was allowed to vary (i.e. participants were able to use at their own pace, ad libitum, as it would occur on an “everyday life” setting). There were no co-interventions, no supplementary modes of delivery, nor usage of prompts (i.e. emails, phone calls, SMS) to use the app."

5-viii) Mode of delivery, features/functionalities/components of the intervention and comparator, and the theoretical framework
"Commercially available apps targeting fitness were identified during previous work evaluating the most popular downloaded apps in NZ [37]. The control group was compared against two other groups, each using an app to promote fitness available on both of the major platforms’ app stores (iTunes and Play Store). The two intervention groups allowed comparison of an identical delivery approach (i.e. stand-alone app) but with distinct design features: 1) use of an immersive app, or 2) use of a non-immersive app. Both apps consisted of a fully automated eight-week training program designed to improve fitness and ability to run 5 km; however, the immersive app featured a game-themed design whereby the training program was embedded with a story where the user is trained to collect supplies and protect a town from zombies. Effective self-regulatory behavior change techniques [38 39] underlie the apps, which allow the users to self-monitor and receive feedback on their training. The apps provided information on running and technique, audio instructions on how to perform the training components, and tracked and displayed progress throughout the program. Usability features of the apps include the ability to workout with music on the device’s library and links to associated websites to interact with other users."

5-ix) Describe use parameters
"Participants were encouraged to use their app three times per week and work their way through each of the workouts, but since this was a pragmatic study, access and usage was allowed to vary (i.e. participants were able to use at their own pace, ad libitum, as it would occur on an “everyday life” setting). There were no co-interventions, no supplementary modes of delivery, nor usage of prompts (i.e. emails, phone calls, SMS) to use the app."

5-x) Clarify the level of human involvement
"There were no co-interventions, no supplementary modes of delivery, nor usage of prompts (i.e. emails, phone calls, SMS) to use the app."

5-xi) Report any prompts/reminders used
"There were no co-interventions, no supplementary modes of delivery, nor usage of prompts (i.e. emails, phone calls, SMS) to use the app."

5-xii) Describe any co-interventions (incl. training/support)
N/A. There were no co-interventions. Apps were used as a stand-alone tool.

6a) CONSORT: Completely defined pre-specified primary and secondary outcome measures, including how and when they were assessed

"The primary outcome was cardiorespiratory fitness, assessed with the one mile run/walk test.Following the procedures outlined in the test administration manual, participants were instructed to run and/or walk at their own pace until completing the distance in the shortest possible time. The output, in seconds, was used to estimate VO2peak using a validated prediction equation.
Secondary outcomes included anthropometrics, self-reported physical activity and associated psychological variables, objectively measured physical activity, and acceptability and usability of the apps. Body weight (in kg, without shoes) was measured with a Salter scale to one decimal point. Height was measured to the nearest 0.1 cm with a Seca stadiometer. Two measurements were taken for each, and the means were used for analysis. BMI was calculated by using the standard equation (weight in kilograms/height in meters squared). Body mass index-for-age was calculated using the WHO growth standards macro.
Using instruments validated in this population, participants self-reported: 1) physical activity using the Physical Activity Questionnaire for Adolescents (PAQ-A), 2) perceived enjoyment using the Physical Activity Enjoyment Scale (PACES) , 3) perceived competence, autonomy and relatedness using the Psychological Need Satisfaction in Exercise Scale (PNSES), 4) self-efficacy using the Physical Activity Self-Efficacy Scale (PASES) , and 5) frequency of app utilization, acceptability and usability of the app via an exit survey conducted with the participants in the intervention groups.
Participants were instructed to wear the accelerometer (Actigraph GT1M) on their right hip during waking hours for seven days after each assessment, removing it when engaging in activities involving water and/or contact sports.
Adverse events were collected at each study visit or voluntarily reported by contacting the researcher. An adverse event was considered serious if required hospitalization."

6a-i) Online questionnaires: describe if they were validated for online use and apply CHERRIES items to describe how the questionnaires were designed/deployed

N/A. No online questionnaires were used.

6a-ii) Describe whether and how "use" (including intensity of use/dosage) was defined/measured/monitored

"Secondary outcomes included (...) self-reported (...) acceptability and usability of the apps."
"Per-protocol analyses were conducted on those participants with complete baseline and follow-up data and self-reported adherence to the training program (i.e. app used 3 times/week)."

6a-iii) Describe whether, how, and when qualitative feedback from participants was obtained

"5) frequency of app utilization, acceptability and usability of the app via an exit survey conducted with the participants in the intervention groups".

6b) CONSORT: Any changes to trial outcomes after the trial commenced, with reasons

"There were no deviations to methods after trial commencement".

7a) CONSORT: How sample size was determined

7a-i) Describe whether and how expected attrition was taken into account when calculating the sample size

"A total of 51 participants (17 per group) was estimated to provide 80% power and α = .05 overall to detect a difference of 17 seconds in cardiorespiratory fitness, assuming a 15 seconds SD in time to complete the one-mile run/walk test, between each of the conditions compared to the control."
Attrition was not taken into account.

7b) CONSORT: When applicable, explanation of any interim analyses and stopping guidelines

N/A, the trial participants were not deemed to be at risk when reviewed by the Ethics Committee.

8a) CONSORT: Method used to generate the random allocation sequence

"A biostatistician (YJ) prepared the randomization scheme in advance by using a computer-generated randomization table."

8b) CONSORT: Type of randomisation; details of any restriction (such as blocking and block size)

"Stratified block randomization in variable blocks was used to maintain balance across sex, an important prognostic factor."

9) CONSORT: Mechanism used to implement the random allocation sequence (such as sequentially numbered containers), describing any steps taken to conceal the sequence until interventions were assigned

"Based on the randomization scheme, a research assistant prepared opaque-sealed envelopes containing group referral so that the researcher could not identify group assignment. The envelope was opened by each subject after completion of baseline assessment".

10) CONSORT: Who generated the random allocation sequence, who enrolled participants, and who assigned participants to interventions

"A biostatistician (YJ) prepared the randomization scheme in advance by using a computer-generated randomization table."
"Enrolled participants (AD) were randomly assigned at a 1:1:1 ratio to one of three conditions."
"Based on the randomization scheme, a research assistant prepared opaque-sealed envelopes containing group referral so that the researcher could not identify group assignment. The envelope was opened by each subject after completion of baseline assessment".

11a) CONSORT: Blinding - If done, who was blinded after assignment to interventions (for example, participants, care providers, those assessing outcomes) and how

11a-i) Specify who was blinded, and who wasn't

"Given the nature of the intervention, it was not possible to blind participants. However, allocation concealment was maintained up to the point of randomization."

11a-ii) Discuss e.g., whether participants knew which intervention was the "intervention of interest" and which one was the "comparator"

"Given the nature of the intervention, it was not possible to blind participants.

11b) CONSORT: If relevant, description of the similarity of interventions

N/A

12a) CONSORT: Statistical methods used to compare groups for primary and secondary outcomes

"Treatment evaluations were performed on the principle of intent-to-treat, including all randomized participants as allocated. Statistical analyses were performed with SAS version 9.4 software (SAS Institute, Cary, NC, USA). All statistical tests were two-sided at a 5% significance level, with adjustment for multiple comparisons on the primary outcome. Analysis of covariance (ANCOVA) regression model was used to evaluate the main treatment effects on the primary outcome, adjusting for baseline measure and sex. Model-adjusted means, 95% CIs and P values were estimated for each group. Mean differences between groups were tested. A similar approach was used for secondary outcomes. Missing data on the primary outcome were imputed with the baseline value. Per-protocol analyses were conducted on those participants with complete baseline and follow-up data and self-reported adherence to the training program (i.e. app used 3 times/week), following the same procedures as in the intention-to-treat analyses."

12a-i) Imputation techniques to deal with attrition / missing values

"Missing data on the primary outcome were imputed with the baseline value."

12b) CONSORT: Methods for additional analyses, such as subgroup analyses and adjusted analyses

"Per-protocol analyses were conducted on those participants with complete baseline and follow-up data and self-reported adherence to the training program (i.e. app used 3 times/week), following the same procedures as in the intention-to-treat analyses."

RESULTS

13a) CONSORT: For each group, the numbers of participants who were randomly assigned, received intended treatment, and were analysed for the primary outcome

"Figure 1 presents the participant flow diagram. Of 143 individuals screened, 51 eligible participants were randomized to the "Zombies, run" immersive app intervention (n=17), "Get running" non-immersive app intervention (n=16), or the control (n=18)".

13b) CONSORT: For each group, losses and exclusions after randomisation, together with reasons

"Figure 1 presents the participant flow diagram".

13b-i) Attrition diagram

N/A
Under the Discussion section, subheading Strengths and Limitations:
"We used readily available apps and consequently were limited to the decisions made by the app developers on content, duration of the program and design features. This also meant that we were unable to access data on app utilization".

14a) CONSORT: Dates defining the periods of recruitment and follow-up

"Recruitment began October 2013 and finished on June 2014. The final follow-up visit was in September 2014".

14a-i) Indicate if critical "secular events" fell into the study period

N/A

14b) CONSORT: Why the trial ended or was stopped (early)

N/A

15) CONSORT: A table showing baseline demographic and clinical characteristics for each group

"Baseline demographic characteristics of participants are presented in Table 1. Participants had a mean age of 15.7 years (14-17 years) and a BMI of 22.9 kg/m2. The majority were NZ European (61%), whereas 22% were Pacific Islanders, and 57% were female."

15-i) Report demographics associated with digital divide issues

"Baseline demographic characteristics of participants are presented in Table 1".
This table lists the participants' devices (i.e. ipod or smartphone brand).

16a) CONSORT: For each group, number of participants (denominator) included in each analysis and whether the analysis was by original assigned groups

16-i) Report multiple "denominators" and provide definitions

"Treatment evaluations were performed on the principle of intent-to-treat, including all randomized participants as allocated."
"Follow-up assessments at 8 weeks were completed for 17 (100%) immersive app group participants, 15 (94%) non-immersive app group participants, and 17 (94%) control group participants".

16-ii) Primary analysis should be intent-to-treat

"Treatment evaluations were performed on the principle of intent-to-treat".

17a) CONSORT: For each primary and secondary outcome, results for each group, and the estimated effect size and its precision (such as 95% confidence interval)

"Table 2 lists the effects of the app interventions on the time to complete the 1-mile walk/run fitness test and all secondary outcomes at 8 weeks. On average time to complete the fitness test decreased in both app groups, but there were no statistically significant differences observed between the intervention groups and the control (-28.4 sec; 95% CI -66.5 to 9.82 sec, P = .20 for the immersive app group; and -24.7 sec; 95% CI -63.5 to 14.2 sec, P = .32 for the non-immersive app group)."
"No intervention effects were found for self-reported secondary outcomes of physical activity (PAQ-A .14; 95% CI -.26 to .54, P = .78 and .23; 95% CI -.18 to .64, P = .42 for the immersive and non-immersive app groups, respectively) or its predictors of perceived enjoyment (PACES), perceived competence, autonomy and relatedness (PNSES), or self-efficacy (PASES) (see Table 2 for all outcomes)."
"For accelerometry, 48 (94%) participants provided valid data for analysis at baseline, whereas compliance with wearing the device slightly decreased at post-intervention (46, 90%). Group assignment did not have a significant effect on overall activity (i.e. average counts per minute) or average daily time spent in moderate-to-vigorous physical activity. Compared to the control, mean baseline daily time spent in MVPA-, sex-, and multiple comparisons-adjusted time in MVPA difference was 1.74 min; 95% CI. -11.45 to 14.93 min, P = .98 and -1.82 min; 95% CI. -16.00 to 12.36 min, P = .99 for the immersive and non-immersive app groups, respectively."

17a-i) Presentation of process outcomes such as metrics of use and intensity of use

"About two-thirds of participants in the intervention groups reported using the app either two (10/32; 31.3%) or three times/week (10/32; 31.3%), whereas 8/32 (25%) only used it one time/week."
"No differences were evident on timing of use (i.e. weekday, weekend, morning, afternoon, evening). Apps were mostly used outdoors (e.g. street, park vs gym, home treadmill) and while alone (n = 13 vs n = 7 with friend and n = 9 with family).
For the app "Zombies, run!", the features mostly used by participants were the "workout mission tasks" (n = 14) and "story & run log" of completed workouts (n = 10), while social networking features ("share my runs" – n = 0 and "ZombieLink account" – n = 3) were seldom used. Results were similar when participants reported the features they liked ("workout mission tasks" = 14, "story and run log" = 8) and disliked ("share my runs" = 5 and "ZombieLink account" = 5).
For the app "Get running", the feature mostly used by participants was the description of the "week – runs" (n = 13), while only one participant reported using the social networking feature "status updates". The description of the "week-runs" was also the feature participants predominantly liked (n = 11), whereas the main feature disliked was the "status updates" (n = 3)."

17b) CONSORT: For binary outcomes, presentation of both absolute and relative effect sizes is recommended

N/A, the primary and secondary outcomes are continuous.

18) CONSORT: Results of any other analyses performed, including subgroup analyses and adjusted analyses, distinguishing pre-specified from exploratory

"In pre-specified per-protocol analyses (i.e. where the app was used 3 times/week), there were statistically significant differences observed on the primary outcome between the non-immersive app group and the control (-79.39 sec; 95% CI -133.01 to -25.77 sec, P = .003). Time to complete the fitness test also decreased for the immersive app group compared to the control, with the difference approaching statistical significance (-55.29 sec; 95% CI -111.46 to .881 sec, P = .055). No intervention effects were found for self-reported secondary outcomes of physical activity, its predictors, nor accelerometry."

18-i) Subgroup analysis of comparing only users

N/A

19) CONSORT: All important harms or unintended effects in each group

"A total of six adverse events (one serious) were reported in six participants, four of which were in the control group (ankle injury – two events, lower back pain, and hospitalization because of tonsils removal) and one in each of the intervention groups (ankle injury – two events). None of the adverse events were deemed related to the study intervention."

19-i) Include privacy breaches, technical problems

N/A

19-ii) Include qualitative feedback from participants or observations from staff/researchers

"Regardless of the app used, similar themes emerged when participants reported their willingness and motives to continue using their app after study participation. Those willing to continue stated personal benefits (e.g. "It will help me to build my fitness", "Because I can improve how far I run") and app related motives (e.g. "A fun way to get fit", "Because it is an enjoyable alternative to exercise"). For those unwilling to continue "Not enough time" was the most common barrier, followed by lack of interest (e.g. "I didn't find the app engaging enough"). The non-immersive app received less positive feedback around motivational aspects (e.g. "Using the app became too tedious").

DISCUSSION

20) CONSORT: Trial limitations, addressing sources of potential bias, imprecision, multiplicity of analyses

20-i) Typical limitations in ehealth trials

"The major limitation of this study was its low statistical power and small sample size. We based our sample size calculation on a smaller standard deviation of the primary outcome than the actual standard deviation observed, which meant the power of the trial was smaller than 80%. We used readily available apps and consequently were limited to the decisions made by the app developers on content, duration of the program and design features. This also meant that we were unable to access data on app utilization (e.g. menus accessed in the app)."

21) CONSORT: Generalisability (external validity, applicability) of the trial findings

21-i) Generalizability to other populations
"We chose a pragmatic approach, where participants used their own device and apps were used ad libitum. Contact with participants was minimal, which reflects app use in a real world context and therefore increases the generalizability of the findings."

21-ii) Discuss if there were elements in the RCT that would be different in a routine application setting
"A major strength of AIMFIT was the use of a randomized controlled trial design to determine the effectiveness of two off-the-shelf commercially available interventions. We chose a pragmatic approach, where participants used their own device and apps were used ad libitum. Contact with participants was minimal, which reflects app use in a real world context and therefore increases the generalizability of the findings."

22) CONSORT: Interpretation consistent with results, balancing benefits and harms, and considering other relevant evidence

22-i) Restate study questions and summarize the answers suggested by the data, starting with primary outcomes and process outcomes (use)
"This is the first randomized trial comparing the effects of a stand-alone immersive mobile app and a non-immersive app on cardiorespiratory fitness, physical activity levels, and its predictors in young people. Key findings were that fitness improved in both app groups, but these did not significantly differ from the control. Despite the availability of commercially readily available apps to improve health behavior, these findings suggest that compared to usual care, no major improvements were found for these two top downloaded apps."

22-ii) Highlight unanswered new questions, suggest future research
"The current one-size-fit all approach of most common commercial mobile apps is limiting for both users and researchers. More tailored approaches, which are dynamic and responsive to changes in PA behavior over time, are required to improve the ease of use of apps, user engagement, and the apps' sustained use."

Other information

23) CONSORT: Registration number and name of trial registry
"The trial was registered (Australian New Zealand Clinical Trials Registry: ACTRN12613001030763) in accordance with the requirements of the International Committee of Medical Journal Editors".

24) CONSORT: Where the full trial protocol can be accessed, if available

"Details on the rationale, design and methods have been previously described [31]".

25) CONSORT: Sources of funding and other support (such as supply of drugs), role of funders
"This is an investigator-initiated study supported by internal funding from the University of Auckland Postgraduate Research Student Support. AD is supported by a Foundation for Science and Technology scholarship (FCT – Portugal SFRH/BD/95762/2013). FCT had no role in experimental design, data collection, or manuscript preparation."

X26-i) Comment on ethics committee approval
"Ethics approval was obtained from the University of Auckland Human Participants Ethics Committee (10054/2013) and the study was conducted according to the principles of the Declaration of Helsinki."

x26-ii) Outline informed consent procedures

"All participants (and guardians for participants younger than 16 years) provided written informed consent."

X26-iii) Safety and security procedures
"Adverse events were collected at each study visit or voluntarily reported by contacting the researcher. An adverse event was considered serious if required hospitalization".

X27-i) State the relation of the study team towards the system being evaluated

"The authors declare that they have no competing interests."
