# Supplementary material for: Apps for IMproving FITness and Increasing Physical Activity Among Young People: The AIMFIT Pragmatic Randomized Controlled Trial
Source: J Med Internet Res. 2015 Aug 27;17(8):e210. doi: 10.2196/jmir.4568 (PMC4642788; doi:10.2196/jmir.4568)
Supplement: Multimedia Appendix 2 [file jmir_v17i8e210_app2.pdf]

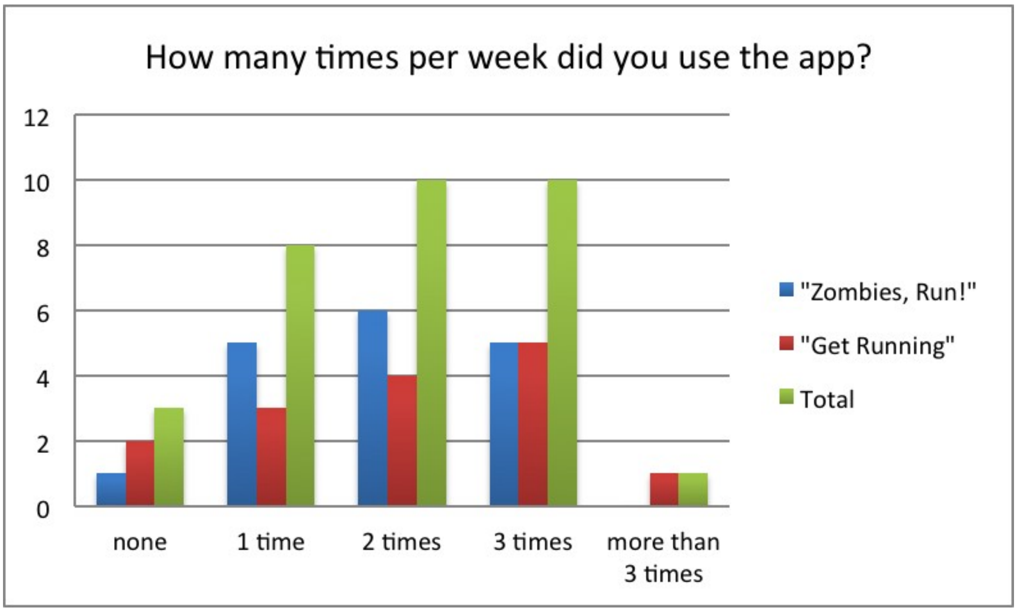
How many times per week did you use the app?
12
10
8
6
4
2
0
none
1 time
2 times
3 times
more than 3 times
"Zombies, Run!"
"Get Running"
Total

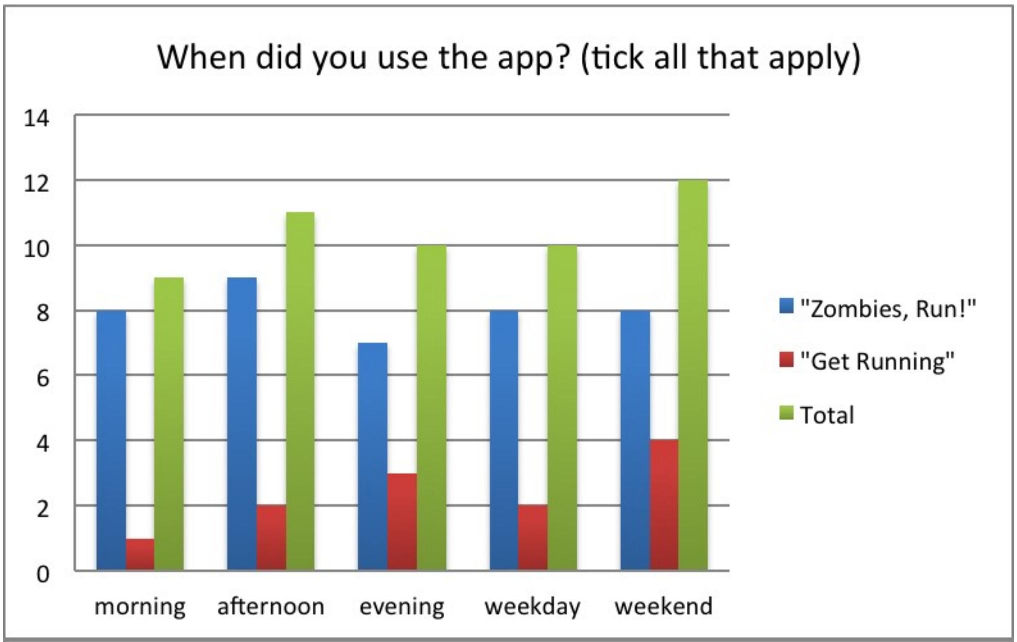
When did you use the app? (tick all that apply)
14
12
10
8
6
4
2
0
morning
afternoon
evening
weekday
weekend
"Zombies, Run!"
"Get Running"
Total

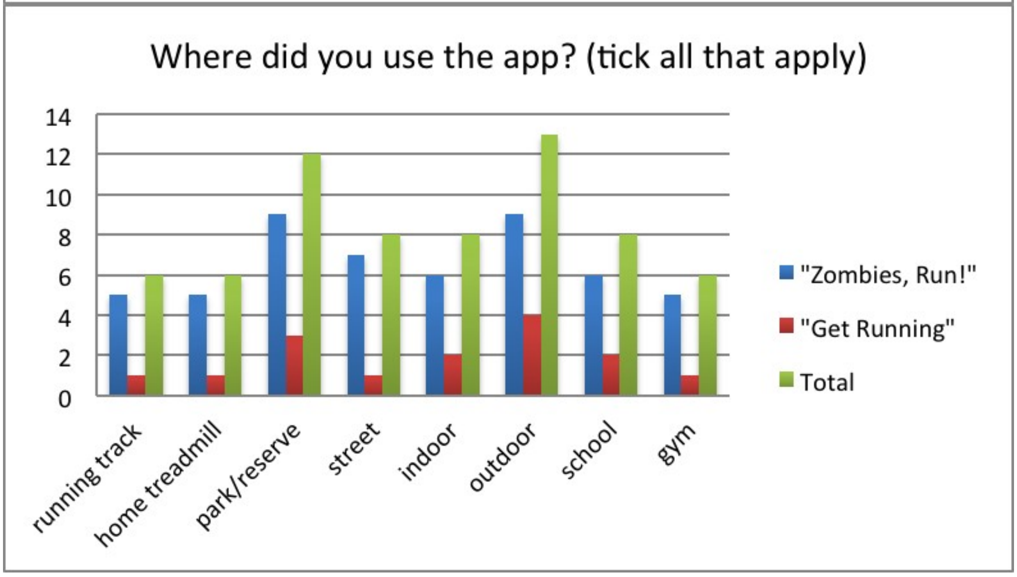
Where did you use the app? (tick all that apply)
14
12
10
8
6
4
2
0
running track
home treadmill
park/reserve
street
indoor
outdoor
school
gym
"Zombies, Run!"
"Get Running"
Total

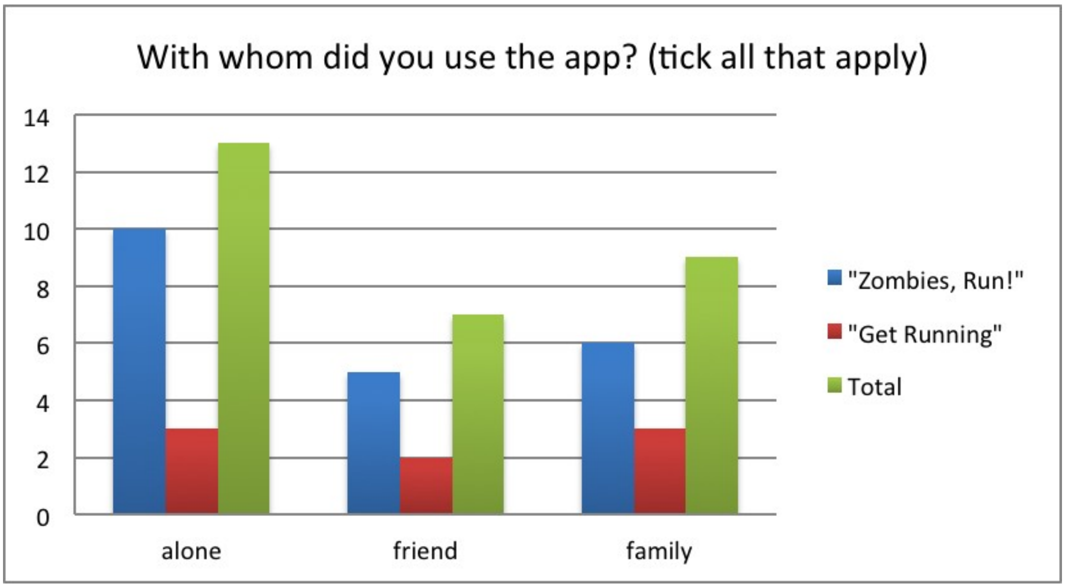
With whom did you use the app? (tick all that apply)
14
12
10
8
6
4
2
0
alone
friend
family
"Zombies, Run!"
"Get Running"
Total

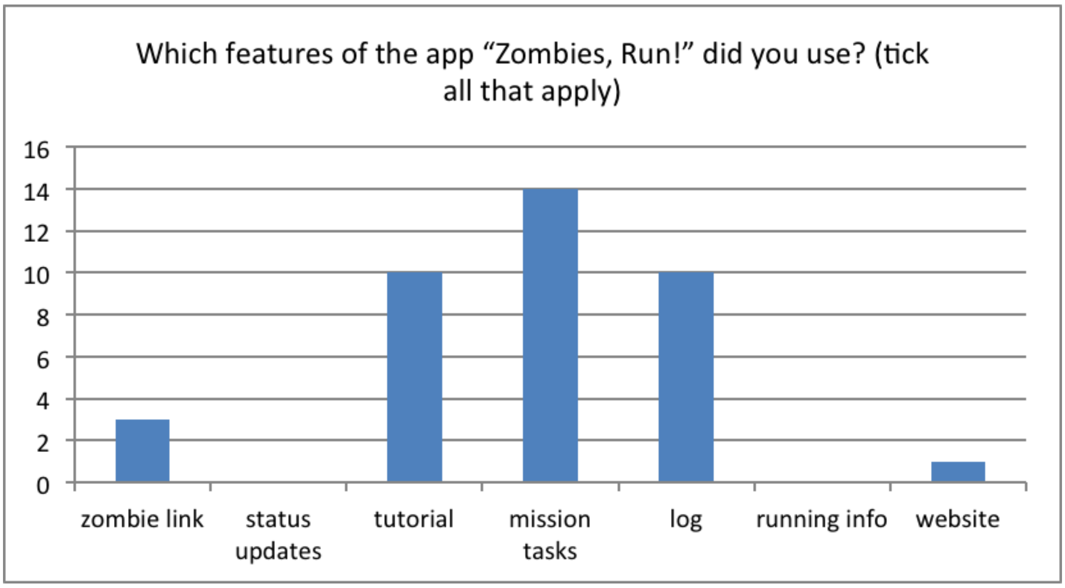
Which features of the app “Zombies, Run!” did you use? (tick all that apply)
16
14
12
10
8
6
4
2
0
zombie link
status updates
tutorial
mission tasks
log
running info
website

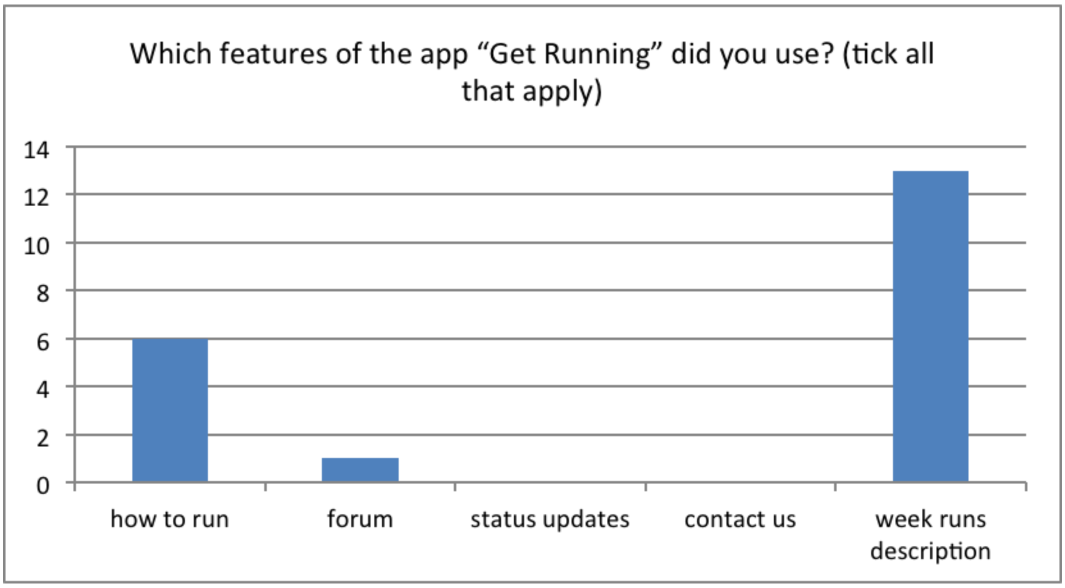
Which features of the app “Get Running” did you use? (tick all that apply)
14
12
10
8
6
4
2
0
how to run
forum
status updates
contact us
week runs description

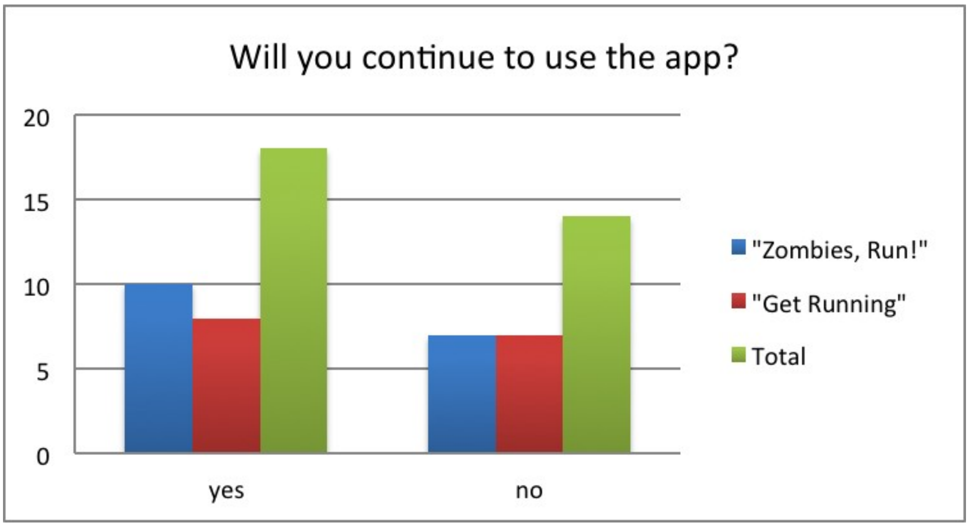
Will you continue to use the app?
20
15
10
5
0
yes
no
"Zombies, Run!"
"Get Running"
Total

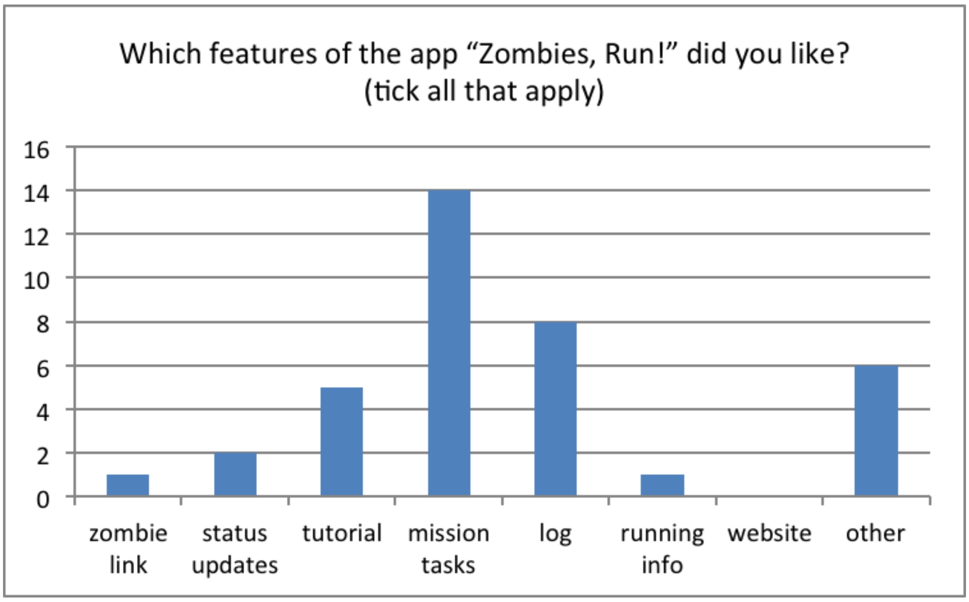
Which features of the app “Zombies, Run!” did you like? (tick all that apply)
16
14
12
10
8
6
4
2
0
zombie link
status updates
tutorial
mission tasks
log
running info
website
other

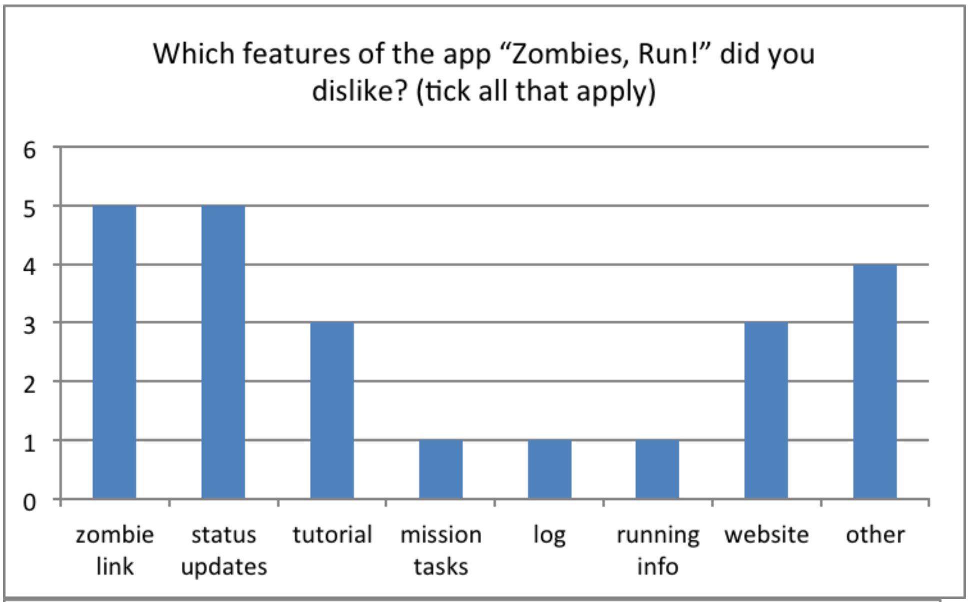
Which features of the app "Zombies, Run!" did you dislike? (tick all that apply)
0
1
2
3
4
5
6
zombie link
status updates
tutorial
mission tasks
log
running info
website
other

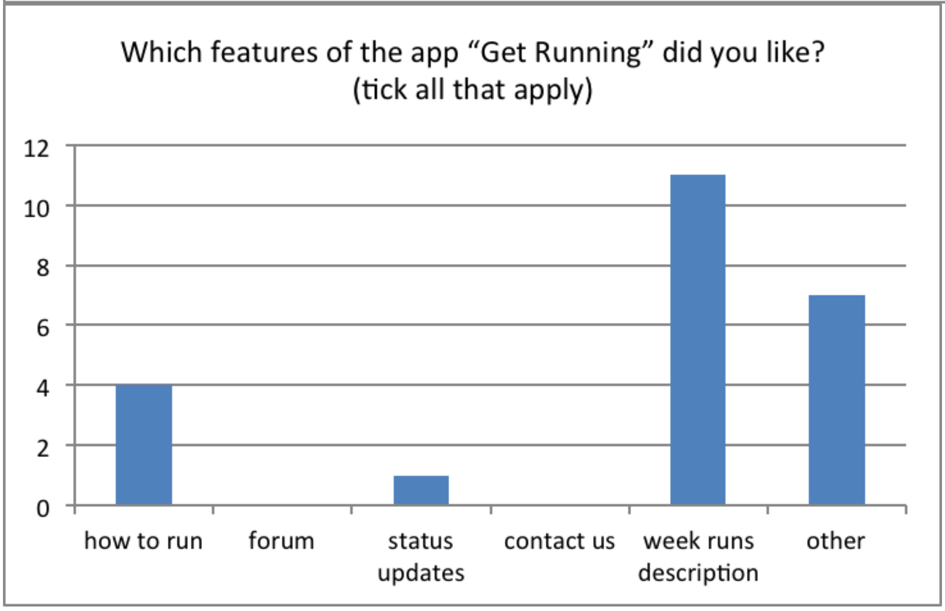
Which features of the app "Get Running" did you like? (tick all that apply)
0
2
4
6
8
10
12
how to run
forum
status updates
contact us
week runs description
other

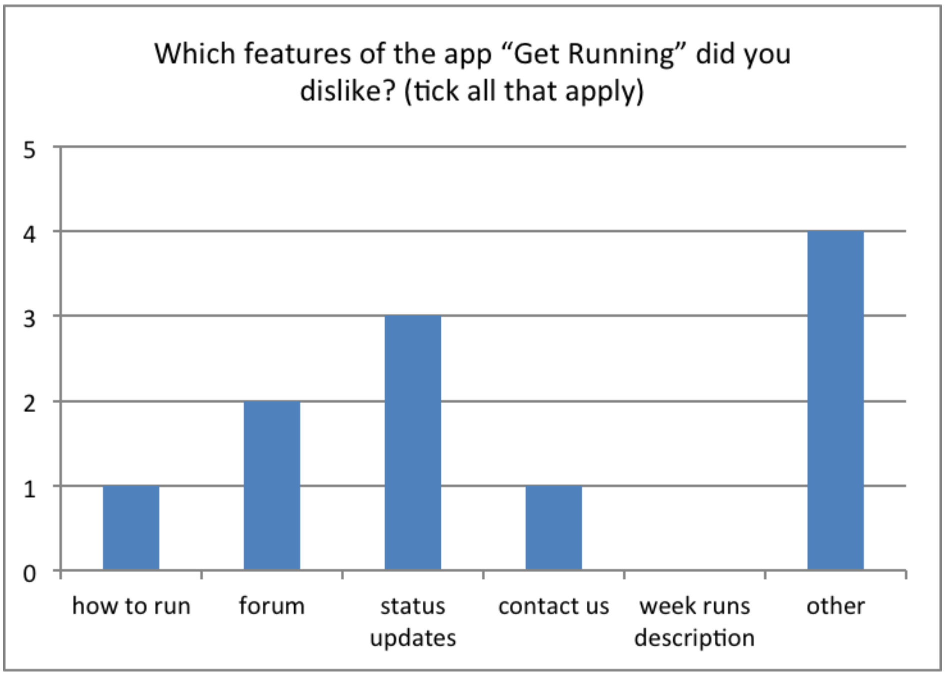
Which features of the app "Get Running" did you dislike? (tick all that apply)
5
4
3
2
1
0
how to run
forum
status updates
contact us
week runs description
other

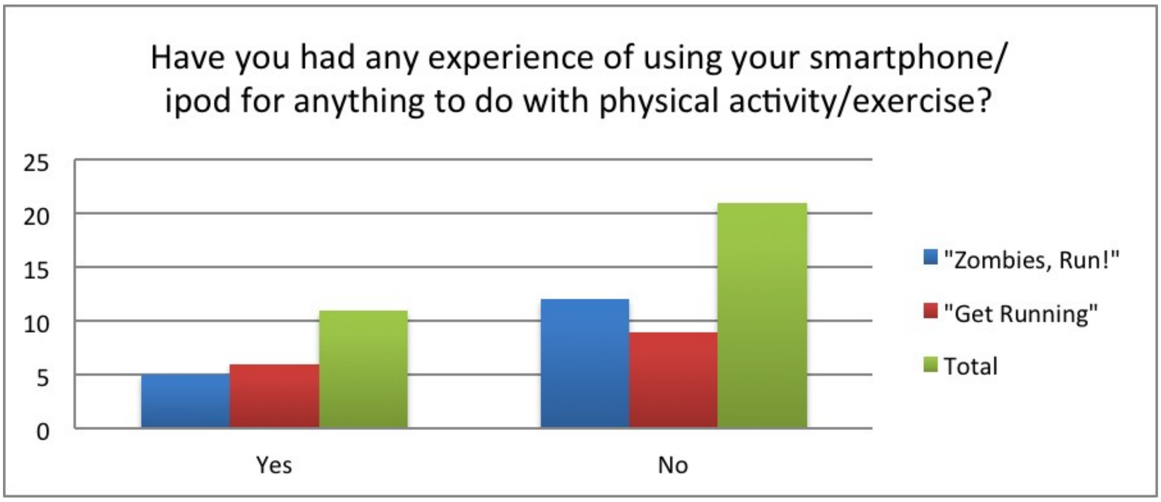
Have you had any experience of using your smartphone/ ipod for anything to do with physical activity/exercise?
25
20
15
10
5
0
Yes
No
"Zombies, Run!"
"Get Running"
Total

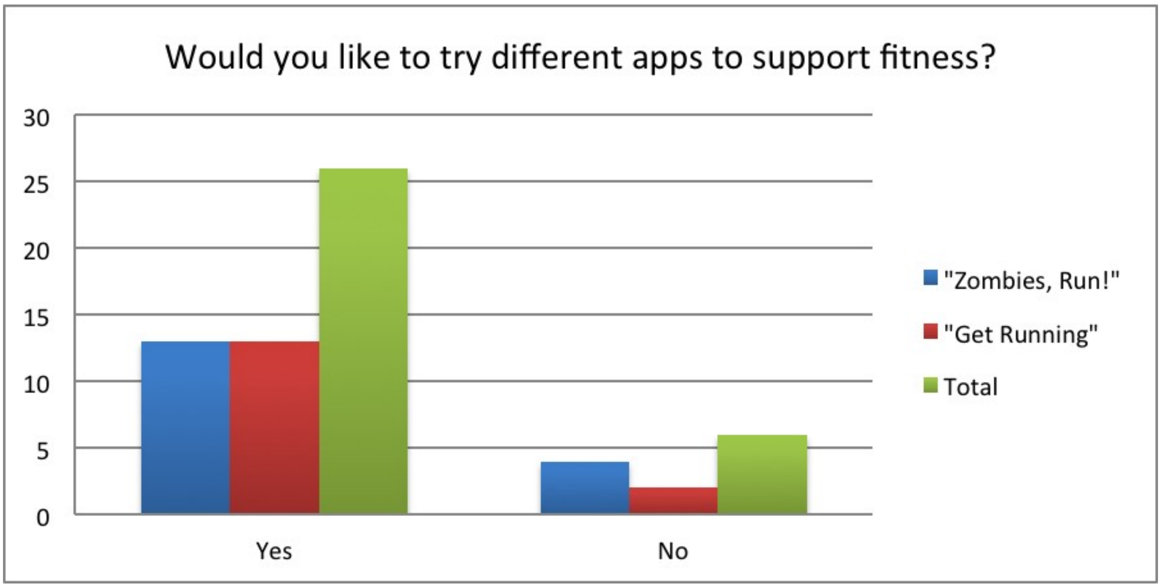
Would you like to try different apps to support fitness?
30
25
20
15
10
5
0
Yes
No
"Zombies, Run!"
"Get Running"
Total
